# P2X7 purinergic receptor modulates dentate gyrus excitatory neurotransmission and alleviates schizophrenia-like symptoms in mouse

**DOI:** 10.1016/j.isci.2023.107560

**Published:** 2023-08-07

**Authors:** Lumei Huang, Paula Mut-Arbona, Bernadett Varga, Bibiana Török, János Brunner, Antonia Arszovszki, András Iring, Máté Kisfali, E. Sylvester Vizi, Beáta Sperlágh

**Affiliations:** 1 Laboratory of Molecular Pharmacology, Institute of Experimental Medicine, 1083 Budapest, Hungary; 2 János Szentágothai Doctoral School, Semmelweis University, 1085 Budapest, Hungary; 3 Laboratory of Cellular Neuropharmacology, Institute of Experimental Medicine, 1083 Budapest, Hungary; 4 Laboratory of Molecular Neurobiology, Institute of Experimental Medicine, 1083 Budapest, Hungary

**Keywords:** Pharmacology, Natural sciences, Biological sciences, Physiology, Neuroscience, Behavioral neuroscience

## Abstract

ATP-gated P2X7 receptors (P2X7Rs) play a crucial role in brain disorders. However, how they affect normal and pathological synaptic transmission is still largely unclear. Here, by using whole-cell patch-clamp technique to record AMPA- and NMDA receptor-mediated excitatory postsynaptic currents (s/mEPSCs) in dentate gyrus granule cells (DG GCs), we revealed a modulation by P2X7Rs of presynaptic sites, especially originated from entorhinal cortex (EC)-GC path but not the mossy cell (MC)-GC path. The involvement of P2X7Rs was confirmed using a pharmacological approach. Additionally, the acute activation of P2X7Rs directly elevated calcium influx from EC-GC terminals. In postnatal phencyclidine (PCP)-induced mouse model of schizophrenia, we observed that P2X7R deficiency restored the EC-GC synapse alteration and alleviated PCP-induced symptoms. To summarize, P2X7Rs participate in the modulation of GC excitatory neurotransmission in the DG via EC-GC pathway, contributing to pathological alterations of neuronal functions leading to neurodevelopmental disorders.

## Highlights

• P2X7R activation increased the DG GC excitatory neurotransmission from EC-GC path
• P2X7R activation directly elevated EC-GC terminals calcium influx
• Genetic ablation of P2X7R restored phencyclidine-induced EC-GC synapse alterations
• P2X7R ablation alleviated phencyclidine-induced behavior changes in mice

## Graphical abstract

**Figure:**
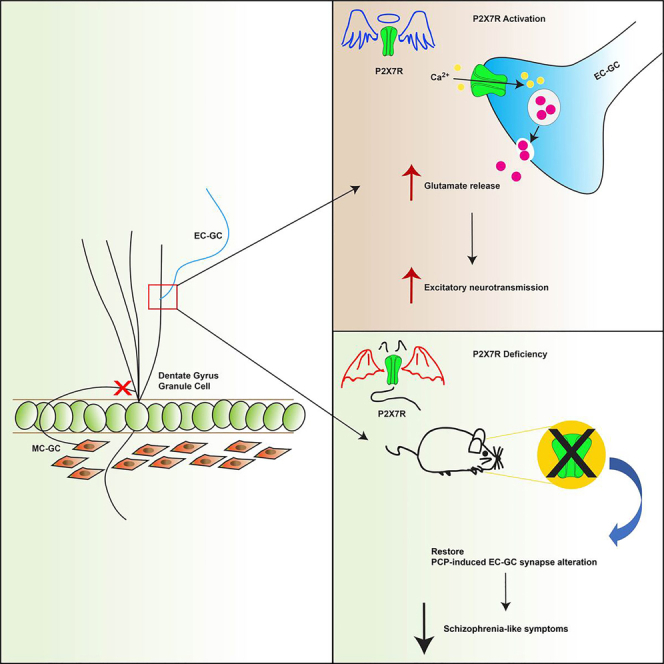

## Introduction

In the central nervous system (CNS), P2X7 receptors (P2X7Rs), the ATP-gated ion channels, are widely expressed in a variety of brain regions, such as the frontal cortex, hippocampus, amygdala, and striatum, and are involved in neurodegenerative diseases and psychiatric disorders.^1^^,^^2^ In view of cell-type specific expression, P2X7Rs are abundantly expressed in microglia and other immune cell types, astrocytes, and oligodendrocytes. Although whether P2X7Rs are expressed in neurons is still a matter of long-standing debate,^3^^,^^4^ P2X7R-positive neurons have been identified in the pyramidal cells in the CA1, CA3, and CA4 regions of the hippocampus and other brain areas in rats using P2X7R mRNA isotopic *in situ* hybridization,^5^ in a humanized mouse P2X7R knock-in model,^6^ and recently in human excitatory but not inhibitory neurons by single-nucleus RNA-sequencing.^7^

To date, studies on the hippocampus have shown a dual effect of P2X7Rs in excitatory neurotransmission in the different subregions of the hippocampus. Early studies detected P2X7Rs immunoreactivity in the axon terminals in the CA1 and CA3 regions. The activation of P2X7Rs by the agonist 2′(3′)-O-(4-benzoylbenzoyl) adenosine 5′-triphosphate triethylammonium salt (BzATP) potentiated the release of glutamate and γ-aminobutyric acid (GABA), effects sensitive to both pharmacological blockade and P2X7R gene deficiency from acute hippocampal slices.^8^^,^^9^ By employing the whole-cell patch clamping technique, another study has demonstrated that activation of presynaptic P2X7Rs enhances the frequency of spontaneous excitatory postsynaptic currents (sEPSCs) in hippocampal hilar neurons.^10^ Recently, a study observed that only CA1 but not CA3 showed the spontaneous postsynaptic currents frequency potentiation in response to BzATP, and this effect is completely abolished by inhibition of astrocyte metabolism with fluorocitrate (FAC).^11^ The main resident cells in the dentate gyrus (DG), granule cells (GC) mainly receive excitatory inputs from the entorhinal cortex (EC) and act as a “gate” in hippocampal trisynaptic neurotransmission. Therefore, it is important to reveal whether P2X7Rs could directly regulate excitatory neurotransmission at this site.

Compelling evidence showing that administration of the N-methyl-D-aspartate (NMDA) antagonists ketamine and phencyclidine (PCP) results in schizophrenic-like symptoms has been obtained to support the glutamate hypothesis of schizophrenia (SZ) psychopathology.^12^ Recently, the largest GWAS study done ever on schizophrenic subjects identified gene polymorphisms of P2X7R associated with the disorder.^13^ Remarkably, acute and subacute PCP-induced SZ symptoms are alleviated by pharmacological inhibition and genetic deletion of P2X7Rs in mice.^14^^,^^15^ Meanwhile, postnatal PCP administration has been employed to establish a neurodevelopmental model of SZ.^16^ A recent study found that acute inhibition of P2X7Rs by the brain-penetrating P2X7R antagonist JNJ-4795567 significantly reverses postnatal PCP-induced impairment of spatial learning and memory as well as locomotor hyperactivity in adults.^17^ Therefore, the impact of P2X7R activity in SZ rodent models have repeatedly demonstrated; yet, the potential underlying mechanism has remained largely unclear. In contrast to pharmacological inhibition of receptors, genetic deficient of P2X7Rs alters the gene expression of glutamate subunits and SZ-related genes in an age- and region-dependent manner. For example, P2X7R ablation increases Grin2b mRNA levels in the adult prefrontal cortex and Grin2a mRNA levels in the juvenile hippocampus.^14^ Thus, it is worthwhile to examine the effect of P2X7R deficiency on postnatal PCP-induced behavioral alterations and the potential mechanism.

In this study, we demonstrated for the first time that P2X7Rs modulate the excitatory neurotransmission in DG GC from the entorhinal cortex-granule cell (EC-GC) pathway, but not from the mossy cell-granule cell (MC-GC) pathway. By directly infecting EC-GC with pAAV1-hSynapsin1-axon-GCaMP6s, we provided further evidence that direct activation of P2X7Rs was sufficient to elevate EC-GC axonal bouton calcium influx. In the postnatal PCP-induced animal model of SZ, we found that the genetic ablation of P2X7Rs restored synaptic plasticity of EC-GC and attenuated PCP-induced memory deficient in juvenile mice. Furthermore, genetic absence of P2X7Rs reversed postnatal PCP-induced behavior alteration in adult mice, indicating the potential therapeutic effect on SZ.

## Results

### P2X7Rs modulate excitatory neurotransmission in both an AP-dependent manner and AP-independent manner

**Figure 1:**
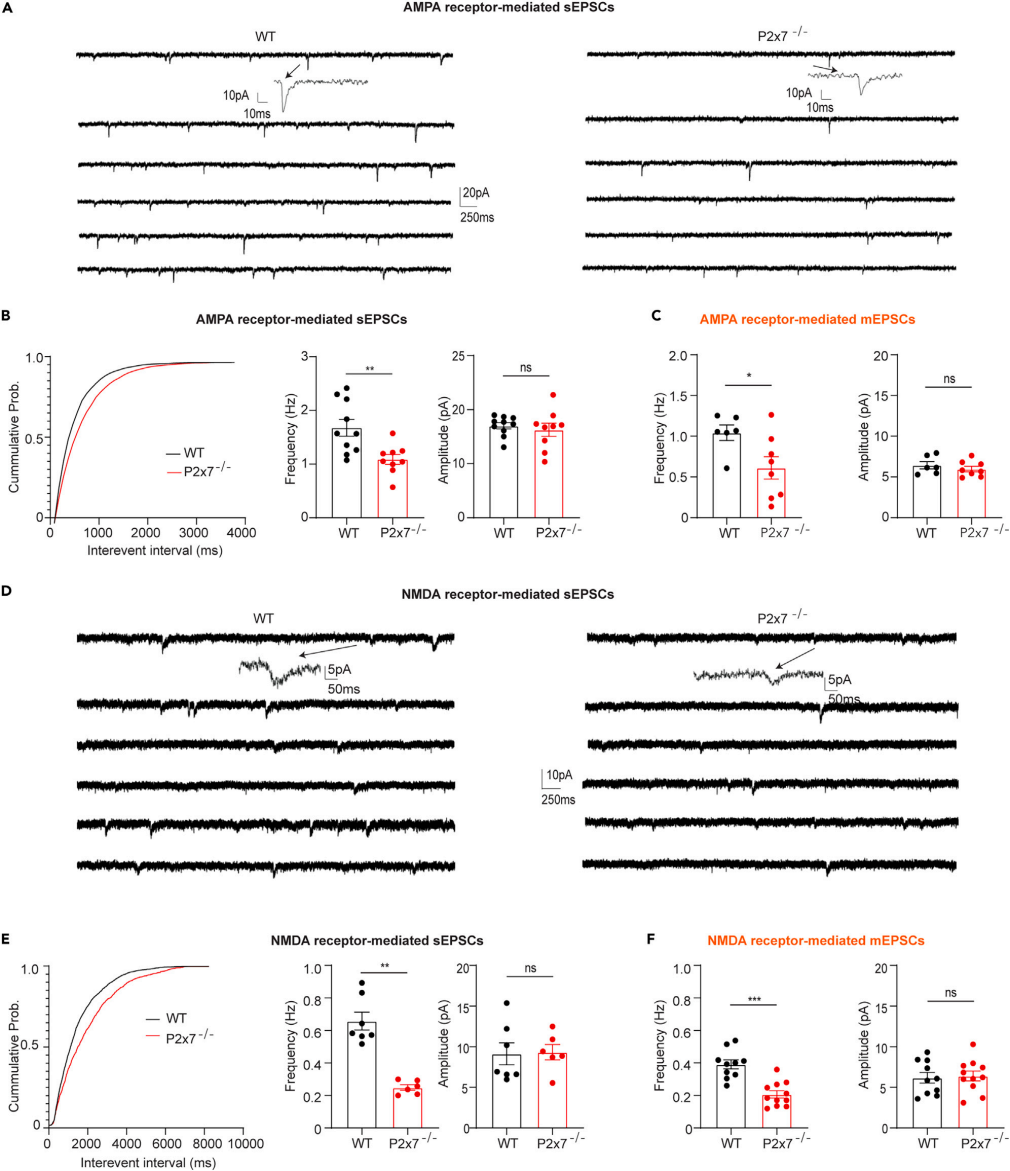
P2X7Rs modulate AMPA- and NMDA receptor-mediated excitatory neurotransmission in both an AP-dependent manner and AP-independent manner (A and B) Representative AMPA receptor-mediated sEPSC recordings from WT (left) and P2X7R deficient (right) mice at P21-28. (B, left) Cumulative distribution of the AMPA receptor-mediated sEPSCs frequency, summary of the frequency (B, middle), and amplitude (B, right) data showing that the frequency decreased while the amplitude was unchanged in P2X7R deficient mice compared to WT mice (Frequency wt: 1.68 ± 0.15 Hz, n = 10 vs. P2x7^−/−^: 1.09 ± 0.09 Hz, n = 9; p = 0.005, unpaired t test: Amplitude wt: 17.06 ± 0.5 pA, n = 10 vs. P2x7^−/−^:17.26 ± 0.59 pA, n = 9; p = 0.58, unpaired t test). (C) Summary of the frequency (left) and amplitude (right) from AMPA receptor-mediated mEPSCs showing the decrease in frequency in P2X7R deficient mice compared to WT mice (Frequency wt: 1.04 ± 0.09 Hz, n = 6 vs. P2x7^−/−^: 0.61 ± 0.14 Hz, n = 8; p = 0.033, unpaired t test; Amplitude wt: 6.44 ± 0.45 pA, n = 10 vs. P2x7^−/−^-: 5.98 ± 0.33 pA, n = 8; p = 0.89, unpaired t test). (D and E) Representative NMDA receptor-mediated sEPSC traces from WT (left) and P2X7R deficient (right) mice at P21-28. (E, left) Cumulative distribution of the NMDA receptor-mediated sEPSC frequency, summary of the frequency (E, middle), and amplitude (E, right) data displaying a decrease in frequency in P2X7R deficient mice in comparison with WT mice (Frequency wt: 0.66 ± 0.06 Hz, n = 7 vs. P2x7^−/−^: 0.25 ± 0.02 Hz, n = 6; p = 0.001, unpaired t test; (Amplitude wt: 9.12 ± 1.36 pA, n = 7 vs. P2x7^−/−^: 9.32 ± 0.96 pA, n = 6; p = 0.83, unpaired t test). (F) Summary of the frequency (left) and amplitude (right) from NMDA receptor-mediated mEPSCs showing the decrease in frequency in P2X7R deficient mice when compared to the WT counterparts (Frequency wt: 0.39 ± 0.027 Hz, n = 10 vs. P2x7^−/−^: 0.19 ± 0.02 Hz, n = 11; p = 0.001, unpaired t test; Amplitude wt: 6.18 ± 0.66 pA, n = 10 vs. P2x7^−/−^: 6.4 ± 0.6 pA, n = 11; p = 0.96, unpaired t test). The cumulative probability was assessed using the Kolmogorov-Smirnov test. Summary data are shown as the mean ± SEM. ∗ marks significant difference.

To address whether P2X7Rs regulate DG GC excitatory neurotransmission, we examined α-amino-3-hydroxy-5-methyl-4-isoxazole propionic acid (AMPA) receptor-mediated sEPSCs and miniature excitatory postsynaptic currents (mEPSCs) in both WT and P2X7R deficient mice at P21-28. To differentiate AMPA receptor-related events, we applied SR 95531 and DL-2-amino-5-phosphonovaleric acid (DL-AP5) to block γ-aminobutyric acid receptor A (GABA_A_) and NMDA receptors, respectively. In contrast to WT mice, P2X7R deficient mice showed less events without alterations in the amplitude (Figures 1A and 1B). Then, we recorded AMPA receptor-mediated mEPSC by adding sodium channel blockers tetrodotoxin (TTX) into SR 95531 and DL-AP5 containing artificial cerebrospinal fluid (ACSF) together with N-ethyllidocaine bromide (QX314) in internal solution to block sodium channels. Similarly, ablation of P2X7Rs induced less frequency but the amplitude was unaffected in mice of the two genotypes (Figure 1C). Taken together, these results suggest that P2X7Rs modulate AMPA receptor-related neurotransmission by regulating the frequency but not the amplitude of currents in an action potential (AP)-dependent and independent manner.

We next recorded NMDA receptor-mediated sEPSCs and mEPSCs in mice of both genotypes. We used Mg^2+^-free ACSF because NMDA receptors are inhibited in a voltage-dependent manner in the presence of Mg^2+^. In addition, NMDA co-agonist D-serine together with GABA_A_ antagonist SR 95531 and AMPA antagonist 6-cyano-7-nitroquinoxaline-2,3-dione (CNQX) were added to the Mg^2+^-free ACSF. A decrease in the frequency but no change in the amplitude of NMDA receptor-mediated sEPSCs was observed in the P2X7R deficiency group compared to the WT group (Figures 1D and 1E), which was consistent with the effect on AMPA receptor-mediated sEPSCs.

**Figure 2:**
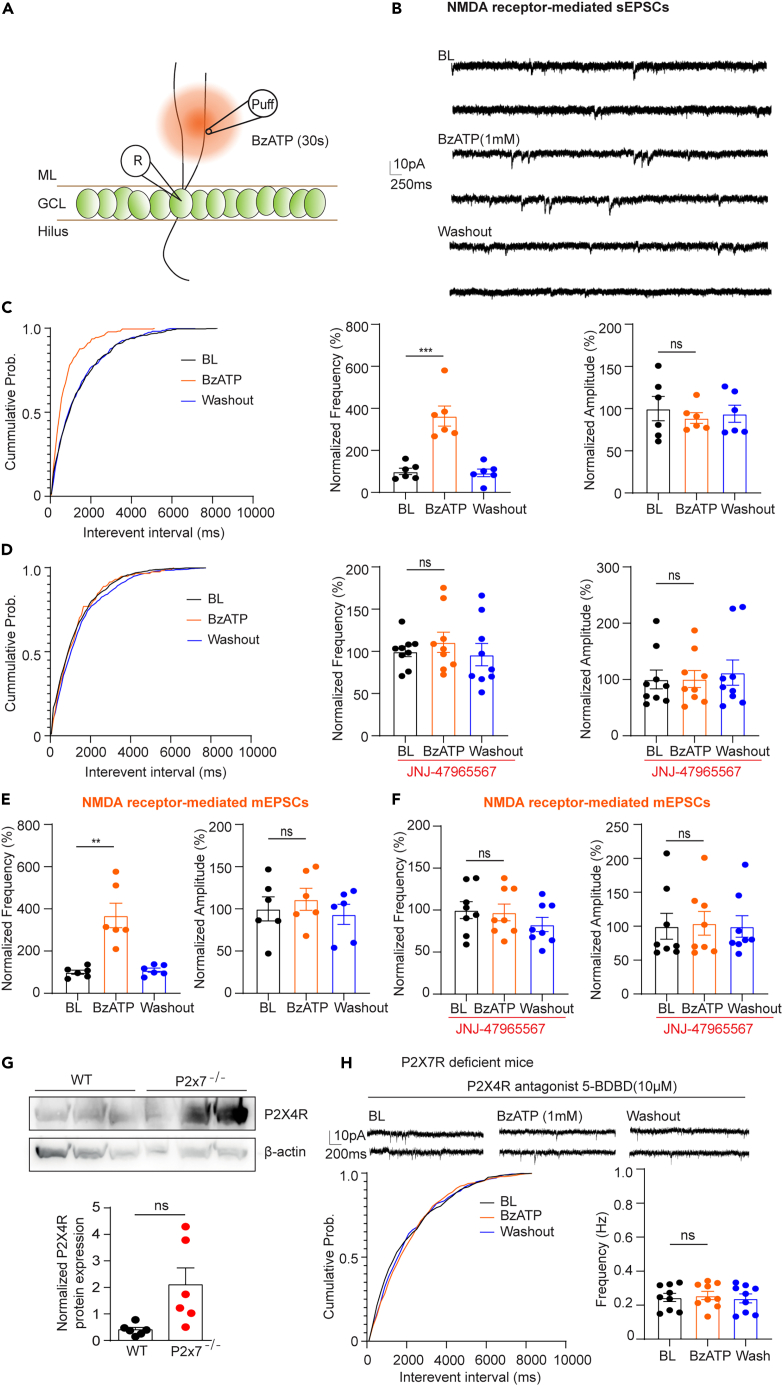
Pharmacological manipulation of P2X7Rs modulates NMDA receptor-mediated excitatory neurotransmission in both an AP-dependent manner and AP-independent manner (A) Schematic of the experimental setup. (B–D) Representative sEPSC traces before (upper two), during (middle two), and after BzATP application (lower two). (C, right) Cumulative probability of sEPSCs interevent intervals and (C, middle and left) normalized frequencies and amplitudes before, during, and after BzATP application. BzATP application increased the number of events but not the amplitude (Normalized frequency (%): baseline: 99.9 ± 15.22; BzATP: 363.39 ± 47.52; Washout: 92.39 ± 28.27; n = 6; baseline vs. BzATP, p < 0.001, one-way ANOVA repeated measures by Dunnett’s test). (D, right) Cumulative probability of sEPSCs interevent intervals and (D, left) normalized frequencies and amplitudes before, during, and after BzATP application in the presence of the selective P2X7R antagonist JNJ-47965567. The increase in frequency was reversed by inhibition of P2X7Rs (Normalized frequency (%): baseline: 94.32 ± 6.83; BzATP: 106.71 ± 12.44; Washout: 108.22 ± 18.11; n = 9; baseline vs. BzATP, p = 0.73, one-way ANOVA repeated measures by Dunnett’s test). (E and F) BzATP increased the frequency of NMDA receptor-mediated mEPSCs (Normalized frequency (%): baseline: 100 ± 9.95; BzATP: 368.99 ± 57.94; Washout: 109.39 ± 10.02; n = 6; baseline vs. BzATP, p = 0.01, one-way ANOVA repeated measures by Dunnett’s test), and (F) this increase was inhibited in the presence of JNJ-47965567 P2X7Rs (Normalized frequency (%): baseline: 100 ± 10.06; BzATP: 97.33 ± 9.99; Washout: 82.89 ± 8.57; n = 7; baseline vs. BzATP, p = 0.73, one-way ANOVA repeated measures by Dunnett’s test). (G) P2X4R protein expression from mouse hippocampi lysates from WT and P2X7R-deficient animals were determined by immunoblotting. Graphs show the densitometric evaluation (n = 6; unpaired t test). The whole blot instead of bands was cropped, since the cropped part held no value (no signal or anything is shown on the rest). All protein bands are shown on the cropped Western blot. (H) NMDARs-sEPSC in the presence of P2X4R selective antagonist 5-BDBD in P2X7R deficient mice. 5-BDBD application reverted the BzATP-induced frequency potentiation (baseline: 0.245 ± 0.02, BzATP: 0.257 ± 0.024, Washout: 0.24 ± 0.03, baseline vs. BzATP, p = 0.92, one-way ANOVA repeated measures by Dunnett’s test). The Kolmogorov-Smirnov test was used to test the significance of cumulative probability. The normalized frequencies and amplitudes are displayed as the mean ± SEM. ∗ marks significant difference.

In line with the findings related to NMDA receptor-mediated sEPSCs, P2X7R deficiency showed less frequency of NMDA receptor-mediated mEPSCs recorded by adding sodium channel blockers TTX and QX314 to block action potential as described previously (Figure 1F). The events were abolished when applied to NMDA antagonist DL-AP5, suggesting that the detected events were mediated by NMDA receptors (Figure S1A). To further confirm the involvement of P2X7Rs in this process, we directly applied the potent P2X7R agonist BzATP via a fast drug application system to the slices in the presence of ARL67156, which protects BzATP from hydrolysis into Bz-adenosine.^18^^,^^19^ We found that activation of P2X7Rs with 1 mM BzATP (Figures 2A–2C) but not 300 μM BzATP (Figures S1C–S1D) increased the frequency of NMDA receptor-mediated sEPSCs. Furthermore, this increase in frequency was efficiently suppressed by bath perfusion of JNJ-47965567, a selective P2X7R antagonist (Figure 2D). Meanwhile, we also found that BzATP increased the frequency of NMDA receptor-mediated mEPSCs (Figure 2E) and the increase was reversed in the presence of JNJ-47965567 (Figure 2F). Next, we asked whether P2X7R-mediated modulation of NMDA receptor-mediated neurotransmission observed in juvenile mice is conserved in adult mice. We examined NMDA receptor-mediated mEPSCs at adult and observed that the frequency of NMDA receptor-mediated mEPSCs was decreased but the amplitude was unaffected in adult P2X7R-deficient mice compared with adult WT mice (Figure S1B). Nevertheless, there was no difference in the frequency of NMDA receptor-mediated mEPSCs between adults and juveniles (Figure S1B).

Although we used P2X7Rs selective antagonist, other P2 receptors could be activated as a result of high concentration of BzATP used. Indeed, we also found that 1 mM BzATP also elevated the NMDA receptor-mediated sEPSCs frequency in P2X7R deficient mice (Figure S1E), although to a lower extent than in WT mice. Given that P2X7Rs could form the heteromeric receptors with other BzATP-sensitive receptors, including P2X4Rs, known to regulate synaptic neurotransmission,^20^^,^^21^ and genetic deficiency of P2X7Rs upregulated the expression of P2X4Rs,^22^^,^^23^^,^^24^ we investigated the involvement of P2X4Rs. By using Western blot, we found that P2X4R protein expression increased in P2X7R deficient mice compared to WT counterparts (Figure 2G). Furthermore, P2X4R selective antagonist 5-BDBD application abolished BzATP-induced NMDA receptor-mediated sEPSC frequency potentiation in P2X7R deficient mice (Figure 2H), suggesting the potential participation of P2X4Rs.

Taken together, the data from both genetic and pharmacological studies suggest that P2X7Rs participate in the regulation of excitatory neurotransmission by altering the frequency of currents and that the regulatory process occurs in both an AP-dependent manner and AP-independent manner.

### P2X7Rs affect excitatory neurotransmission through the EC-GC pathway but not MC-GC pathway

**Figure 3:**
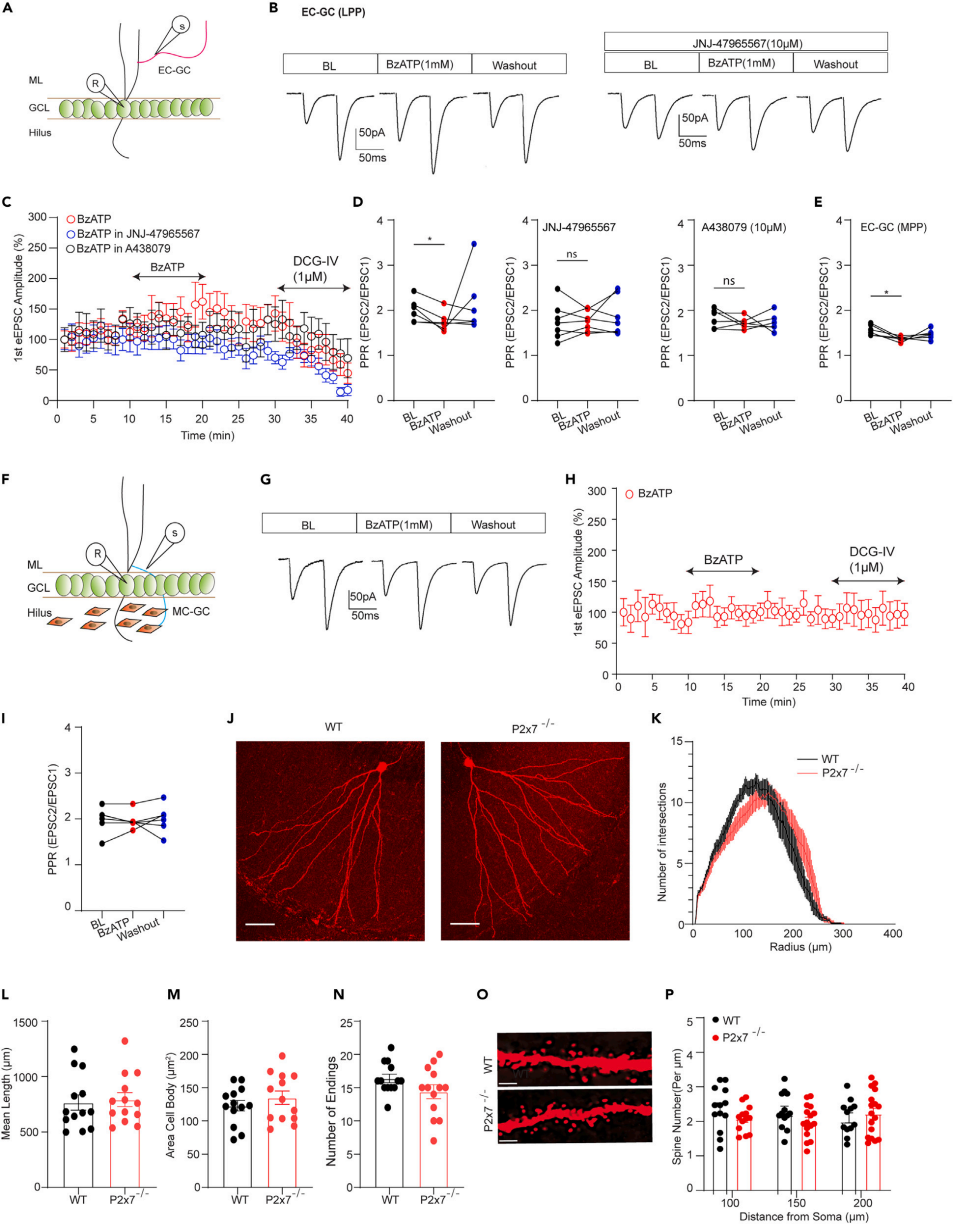
P2X7Rs affect excitatory neurotransmission through the EC-GC pathway but not MC-GC pathway (A) Stimulation pattern showing the position of the stimulating and recording electrodes. (B) The representative traces before, during, and after BzATP application in the presence of 1.3 mM Ca2+/2 Mm Mg2+. (C and D) The 1st stimulus-induced current amplitude changed cross time. DCG-IV perfusion significantly decreased the current amplitude. (D, left) BzATP alone significantly decreased the PPR (PPR: baseline: 2.00 ± 0.09; BzATP: 1.75 ± 0.07; Washout: 2.16 ± 0.23; n = 6; baseline vs. BzATP, p = 0.0249, one-way ANOVA repeated measures by Dunnett’s test). (D, middle) BzATP-induced PPR elevation was inhibited by JNJ-4796567 (PPR: baseline: 1.75 ± 0.15; BzATP: 1.75 ± 0.0.08; Washout: 1.85 ± 0.16; n = 7; baseline vs. BzATP p = 0.99, one-way ANOVA repeated measures by Dunnett’s test). (D, right) BzATP-induced PPR change was also inhibited by another P2X7Rs antagonist A 438079 (PPR: baseline: 1.82 ± 0.08; BzATP: 1.72 ± 0.05; Washout: 1.72 ± 0.08; n = 6; baseline vs. BzATP p = 0.44, one-way ANOVA repeated measures by Dunnett’s test). (E) The BzATP-induced reduction of PPR also showed in MPP (PPR: baseline: 1.56 ± 0.05; BzATP: 1.36 ± 0.02; Washout: 1.47 ± 0.04; n = 6; baseline vs. BzATP p = 0.02, one-way ANOVA repeated measures by Dunnett’s test). (F) Stimulation pattern for the MC-GC pathway. (G) The representative traces before, during and after BzATP application in the presence of 1.3 mM Ca2+/2 Mm Mg2+. (H) BzATP application and DCG-IV administration did not change 1st stimulus-induced current amplitude. (I) BzATP application did not alter PPR in the MC-GC pathway (PPR: baseline: 1.98 ± 0.09; BzATP: 1.96 ± 0.07; Washout: 2.00 ± 0.12; n = 6; baseline vs. BzATP, p = 0.56, one-way ANOVA repeated measures by Dunnett’s test). (J) Two representative DG GCs from WT and P2X7R deficient mice (objective: 20x; scale bar: 50 μm). (K–N) Dendritic morphology of DG GCs. The number of intersections, dendritic length, Area cell body and number of endings were not different between P2X7R deficient mice and WT mice (mean length (μm): wt: 756.57 ± 68.56, n = 13 vs. P2x7−/−:792.64 ± 62.71, n = 13, p = 0.92, unpaired t test; Area Cell Body (μm^2^): wt: 122.83 ± 7.99, n = 13 vs. P2x7−/−: 134.84 ± 1.00, n = 13, p = 0.23, unpaired t test; Number of Endings: wt: 16.38 ± 0.65, n = 13 vs. P2x7−/−: 14.38 ± 1.05, n = 13, p = 0.6, unpaired t test). (O) Two dendritic branches from 150 μm segment (objective: 60x; scale bar: 10 μm). (P) Quantification of the spine number per μm was not significantly different between mice of the two genotypes (n = 13–17). The data are presented as the mean ± SEM. ∗ marks significant difference.

Although DG GCs receive excitatory inputs from entorhinal cortex (EC-GC), the hilar mossy cell (MC-GC)^25^ as well as the supramammillary nucleus,^26^^,^^27^ we mainly focused on whether P2X7Rs regulated excitatory neurotransmission in an input specific way in EC-GC and MC-GC pathway. To this end, we specifically stimulated the two pathways by placing a stimulating electrode into the lateral and medial molecular layer of the dentate gyrus (DG), which contain EC-GC pathway fibers (lateral perforant path [LPP] and medial perforant path [MPP])^28^ (Figure 3A), or the inner molecular layer of the DG, which contains MC-GC pathway axons (Figure 3F).^25^ Simultaneously, a selective agonist of group II metabotropic glutamate receptors (mGluR2/3), DCG-IV, was used to pharmacologically distinguish the two pathways, as mGluR2/3 is not expressed in the MC-GC pathway,^28^ by recording the paired-pulse ratio (PPR). In the presence of normal ACSF containing 1.3 mM Ca^2+^/2 mM Mg^2+^, we found that DCG-IV decreased the 1^st^ pulse-induced NMDA receptor-amplitude in the EC-GC LPP pathway and EC-GC MPP pathway (Figures 3C and S2F). Intriguingly, 1 mM BzATP significantly decreased the PPR value in LPP (Figures 3B and 3D) and MPP (Figure 3E) and this decrease was reversed by bath application of JNJ-47965567 (Figure 3D, middle) and another selective P2X7R antagonist A 438079 (Figure 3D, right). Moreover, we obtained similar results in the presence of 0.5 mM Ca^2+^/0 mM Mg^2+^. BzATP application also decreased the PPR in low calcium ACSF (Figure S2C), and this decrease was reversed by bath application of JNJ-47965567 (Figure S2C). However, BzATP-induced effect was eliminated when we increased the extracellular calcium concentration to 2.6 mM (Figure S2E). These data suggested that the effect of BzATP was dependent on the extracellular calcium concentration, consistently with the properties of the P2X7R ion channel.

Remarkably, when we placed a stimulating electrode in the outer GC layer, which contains axons of the MC-GC pathway (Figure 3F), DCG-IV perfusion did not change the 1^st^ pulse-induced NMDA receptor-amplitude (Figure 3H). Importantly, BzATP application no longer altered the PPR in normal ACSF containing 1.3 mM Ca^2+^/2 mM Mg^2+^ (Figures 3G and 3I) and in ACSF containing 0.5 mM Ca^2+^/0 mM Mg^2+^ (Figure S2D). These data suggest that the EC-GC pathway but not the MC-GC pathway is associated with P2X7R-mediated modulation of neurotransmission.

Because a decrease in the postsynaptic spine number can also contribute to a lower current frequency, we next examined biocytin-labeled GC dendritic morphology and spine number. We found that the length and number of intersections of DG GC dendrites were not altered in the P2X7R-deficient group compared to the WT group (Figures 3J–3N). Subsequently, quantification of the spine numbers showed that there was no change in spine density per μm of dendrite at distances of 100, 150, and 200 μm from the soma in mice of the two genotypes (Figures 3O and 3P). Previous electrophysiological studies have revealed a potential link between P2X7Rs and NMDARs in pyramidal neurons^14^ and in neural progenitor cells (NPCs) in the subgranular zone of the DG.^11^ However, whether these receptors directly interact in DG GCs soma is unclear. Thus, we applied NMDA at concentrations of 10 and 100 μM for 10 s to evoke NMDARs inward currents (Figure S2G), which were dramatically inhibited by the NMDA antagonist DL-AP5 (Figure S2K). After two stable baseline current responses were recorded, 1 mM BzATP (Figures S2H and S2I) or 10 μM JNJ47965567 (Figures S2H and S2J) was applied. We found that NMDARs currents were unaffected by pharmacological manipulation of P2X7Rs (activation or inhibition), suggesting that there was no interaction between P2X7Rs and NMDARs in the DG GCs soma. Additionally, we also checked whether the genetic absence of P2X7Rs changed the intrinsic excitability of the DG GCs in juvenile and young adult mice. We did not observe the difference in the membrane characteristic and intrinsic excitability when compared to WT mice in juveniles, although the adult mice showed slightly higher action potential amplitude compared to the juveniles in WT but not in P2X7R deficient mice (Table S2). Therefore, the postsynaptic mechanism is unlikely to be associated with P2X7R mediated modulation of excitatory neurotransmission demonstrated above. Overall, our current data demonstrated that P2X7Rs regulated excitatory neurotransmission in DG GCs through the EC-GC pathway but not MC-GC pathway.

### P2X7Rs regulate EC-GC axonal boutons calcium influx

**Figure 4:**
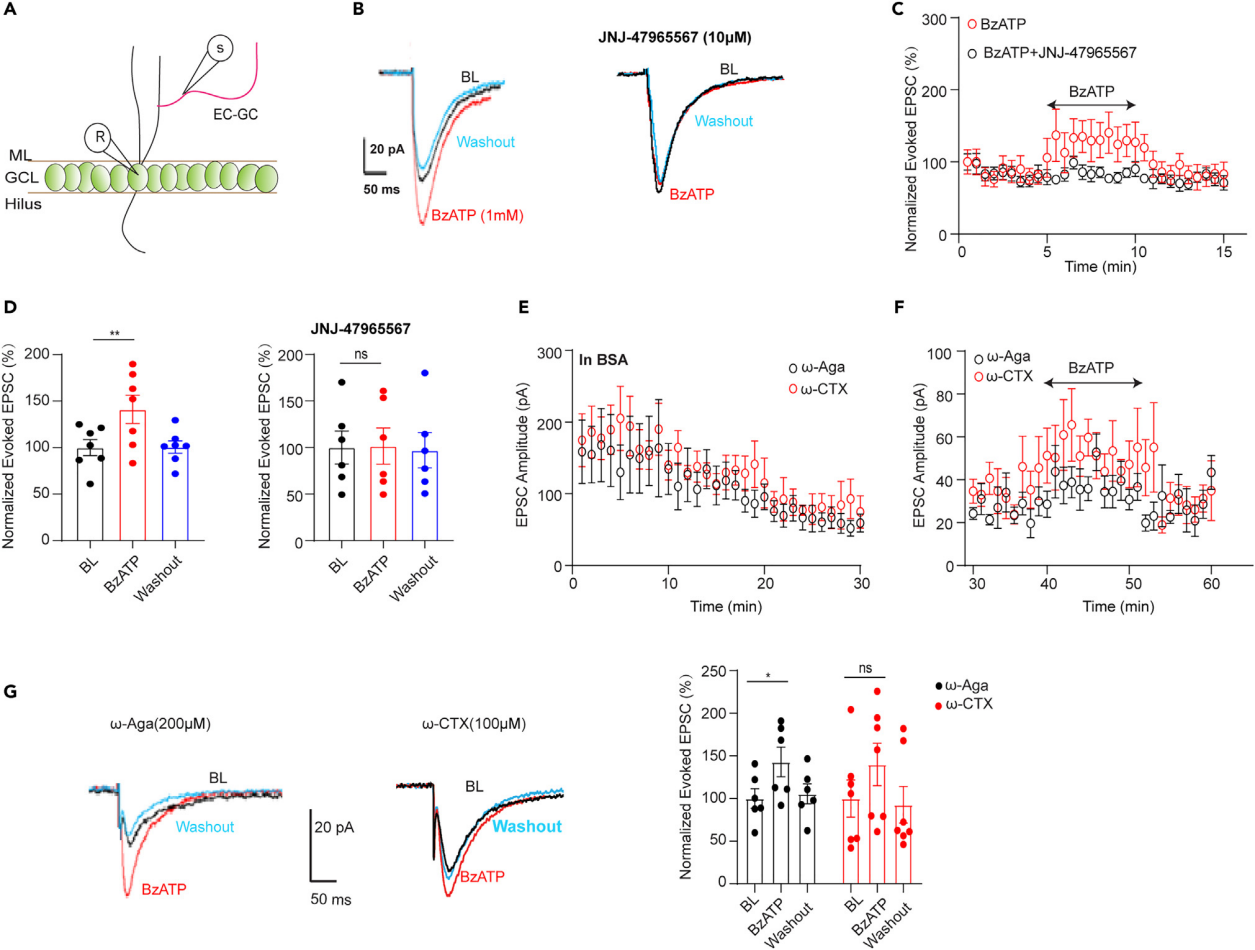
P2X7Rs regulated EC-GC EPSCs independent of N-type and P/Q-type VGCCs (A)The scheme for the stimulation pattern. (B) Representative traces of evoked-EPSCs. (C and D) The Evoked-EPSC change over the time and the summary table. BzATP increased the current amplitude (Normalized EPSC (%): baseline: 100 ± 8.67; BzATP: 141.15 ± 15.23; Washout: 100.59 ± 6.71, n = 7; baseline vs. BzATP, p = 0.027, one-way ANOVA repeated measures by Dunnett’s test) and the increase was inhibited by JNJ-4795567(Normalized EPSC (%): baseline: 100 ± 17.73; BzATP: 101.56 ± 19.43; Washout: 97.03 ± 19.03, n = 7; baseline vs. BzATP, p = 0.97, one-way ANOVA repeated measures). (E) N-type and P/Q-type calcium blockers application decreased the EPSC amplitude in the presence of BSA. (F and G) BzATP application increased EPSC amplitude in the presence of ω-agatoxin IVA (Normalized EPSC (%): baseline: 100 ± 11.6; BzATP: 142.94 ± 17.36; Washout: 105.46 ± 11.66, n = 6, baseline vs. BzATP, p = 0.05, one-way ANOVA repeated measures by Dunnett’s test) or ω-CTX GVIA (Normalized EPSC (%): baseline: 100 ± 21.68; BzATP: 140.08 ± 24.92; Washout: 92.64 ± 21.51, n = 7, baseline vs. BzATP, p = 0.06, one-way ANOVA repeated measures by Dunnett’s test). The data are expressed as the mean ± SEM. ∗ marks significant difference.

Although presynaptic vesicle release is not exclusively dependent on Ca^2+^ entry, we assessed whether P2X7Rs are involved in neurotransmission by modulating presynaptic Ca^2+^ influx. We first stimulated the EC-GC to evoke EPSCs in DG GCs and then applied 1 mM BzATP in the presence of ARL-67156. Consistent with the findings related to the PPR, activation of P2X7Rs increased the amplitude of evoked EPSCs, and this effect was abolished by JNJ-47965567 (Figures 4A–4D). One of the potential underlying mechanisms was the influx of calcium ions directly through P2X7Rs due to their relative permeability to calcium ions. In the perforant path, P/Q-type and N-type VGCCs are the predominant Ca^2+^ sources that trigger neurotransmitter release, although N-type VGCCs show less sensitivity.^29^ Therefore, we applied 200 μM ω-Aga TK, a selective P/Q type calcium channel blocker, or 100 μM ω-CTX GVIA, an N-type calcium channel blocker. We perfused the slice with above two calcium blockers in the presence of BSA. Under these conditions, the EPSC amplitude slowly decreased (Figure 4E). After 30 min’s perfusion, the EPSC amplitude stabilized and we applied to P2X7Rs agonist or antagonist (Figure 4F). We observed an increase in the amplitude of evoked EPSCs in the presence of BzATP. However, a significant BzATP-induced increase in EPSC amplitude was maintained in the presence of P/Q type calcium channel blocker and was slightly maintained after N-type calcium channel blocker application (Figure 4G). These results suggested that P2X7Rs affected EPSC mostly independent of N- and P/Q type calcium channels.

**Figure 5:**
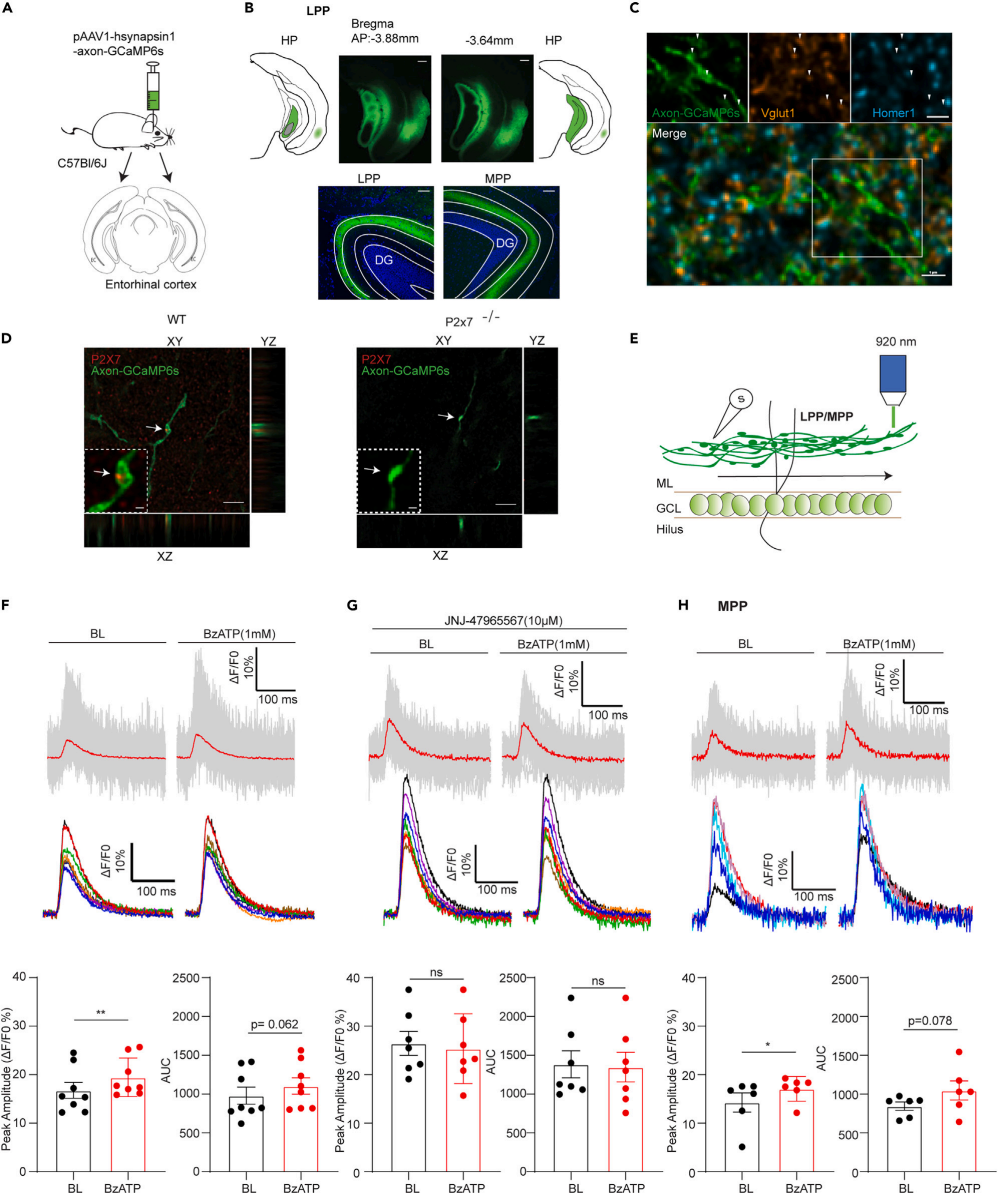
P2X7Rs regulate EC-GC axonal boutons calcium influx (A) The scheme for virus injection. (B, upper) Location of virus injection in the LEC (objective: 4X; scale bar: 200 μm). (B, lower) Virus expression in LPP and MPP (objective: 2X; scale bar: 50 μm). (C) Co-staining GCaMP6s infected fibers with presynaptic marker VGlut1 and postsynaptic marker Homer1 (objective: 60X oil; scale bar: 1 μm). VGlut1 but not Homer1 localized in the GCaMP6s infected axonal boutons. (D) P2X7R immunostaining in WT and P2X7R deficient mice (objective: 60X oil; scale bar: 20 μm and 1 μm). Deconvoluted image showing P2X7R puncta (red dots) on LPP boutons (green) in WT but not in P2X7R deficient mice. The localization of P2X7R puncta on boutons (indicated by arrows) in the XY (big square), YZ (rectangle on the right), and XZ (rectangle on the left) dimensions (objective: 60X oil; scale bar: 5 μm). (E) Schematic of the method used for calcium imaging under a multiphoton microscope. (F) LPP-boutons calcium imaging in the presence of 1.3 mM Ca2+/2 mM Mg2+. Representative boutons calcium traces for one slice (upper) and the average traces from 8 slices (middle). The lower summarized table showed that BzATP application elevated the calcium influx peak amplitude (BL(%DF/F0): 16.75 ± 1.45 VS. BzATP (%DF/F0):19.45 ± 1.24, n = 8; paired t test, p = 0.002) and slightly increased the area under the curve (BL(A.U): 979.86 ± 96.25 VS. BzATP (A.U):1103.85 ± 92.83, n = 8; paired t test, p = 0.062). (G) JNJ-4795567 perfusion inhibited the elevation induced by BzATP (BL(%DF/F0): 26.44 ± 2.14 VS BzATP (%DF/F0):25.35 ± 2.35, n = 7, paired t test, p = 0.15) (BL(A.U): 1383.42 ± 151.24 VS BzATP (A.U):1347.2 ± 164.98, n = 7; paired t test, p = 0.56). (H) MPP-boutons calcium imaging in the presence of 1.3 mM Ca2+/2 mM Mg2+. BzATP application also increased the calcium influx peak amplitude (BL(%DF/F0): 14.28 ± 1.98 VS. BzATP (%DF/F0):17.08 ± 1.03, n = 6, paired t test, p = 0.04) and area under the curve (BL(A.U): 843.8 ± 54.17 VS. BzATP (A.U):1048 ± 123.5, n = 6; paired t test, p = 0.078). The data are presented as the mean ± SEM. ∗ marks significant difference.

To further investigate whether P2X7Rs directly affect the influx of Ca^2+^ into EC-GC axonal boutons, we utilized an axon-targeted genetically encoded calcium indicator (GECI) to avoid contaminating the signals from soma and dendrites. The virus pAAV1-hSynapsin1-axon-GCaMP6s was stereotaxically injected into the lateral entorhinal cortex (LEC) and medial entorhinal cortex (MPP). We found that after two weeks, this novel calcium indicator was successfully targeted to LPP and MPP axons and boutons (Figures 5A and 5B). To further identify the fibers, we co-stained presynaptic marker VGlut1 and postsynaptic marker Homer1. We found that the VGlut1 positive puncta but not Homer1 were localized in the boutons, indicating the fibers were excitatory axons (Figure 5C). Importantly, P2X7R fluorescence puncta were localized on these boutons in WT but not in P2X7R deficient mice (Figure 5D), revealing the expression of P2X7Rs in these excitatory terminals. The quantification data further showed that LPP and MPP boutons did not have difference in the labeling numbers of P2X7Rs puncta (Figure S3A). To further address the functionality of P2X7Rs on boutons, we conducted calcium imaging experiments. We sparsely labeled the LPP with a diluted virus (1:10 in PB buffer). To evaluate the functionality of this indicator in acute hippocampal slices, we electrically stimulated the LPP with an extracellular electrode placed 200 μm away from the scanning area (Figure 5E). To assess the saturation of the indicator, we applied 5 pulses with frequencies of 10, 30, 60, and 100 Hz in random order (60 Hz > 10 Hz > 100 Hz > 30 Hz) under normal ACSF solution containing 1.3 mM Ca^2+^/2 mM Mg^2+^. We found that 5 pulses of 60 Hz led to saturation of the axonal indicator GCaMP6s, since no further increase in the fluorescence intensity in response to a higher frequency was detected (Figures S3B–S3C). Therefore, we utilized 5 pulses of 10 Hz for following experiments. Firstly, we found that BzATP elevated electrical pulse-induced fluorescence intensity (and slightly increased the area under the curve) (Figure 5F). To further confirm that the elevation of calcium influx is tightly linked to P2X7R activation, we administered the selective P2X7R antagonist JNJ47965567. The ATP-induced elevation was inhibited when perfused the slices with P2X7Rs antagonist JNJ47965567 (Figure 5G). We examined the similar results when targeted to MPP (Figures 5E and 5H), suggesting LPP and MPP behaved same. Similarly, we also recorded the P2X7R-mediated bouton calcium influx in ACSF containing 0.5 mM Ca^2+^/0 mM Mg^2+^. BzATP application also increased the calcium spike amplitude compared to baseline (Figure S3E, upper). The control group without BzATP did not change the amplitude indicating the recording system was stable (Figure S3D). Particularly, BzATP application in this condition significantly increased the area under the curve. Again, the increased spike amplitude and the area under the curve were inhibited by JNJ47965567 (Figure S3E lower).

Owing to the evidence that BzATP also increased mEPSC frequency in P2X7Rs knockout mice, we also examined the LPP bouton calcium influx in P2X7R mice. By counting the total number of detected boutons and proportion of boutons which had positive response to BzATP (positive responder), we found that 198 boutons of the total 610 boutons displayed a positive response to BzATP in P2X7R deficient mice. Although the proportion of positive responders in P2X7R deficient mice was slightly higher than in WT-control group (48 of the total 228 boutons) and WT-JNJ47965567+BzATP group (76 of the total 397 boutons), it was still significantly less than in WT-BzATP group (331 of the total 661 boutons) (Figure S3F). Together with the puncta quantification data, these results suggested the heterogeneous expression of P2X7Rs in EC-GC axonal boutons and P2X7R directly regulated calcium influx from P2X7R expressing LPP and MPP axonal boutons.

### P2X7Rs restore EC-GC synapse alteration and alleviate schizophrenia-like behavior

**Figure 6:**
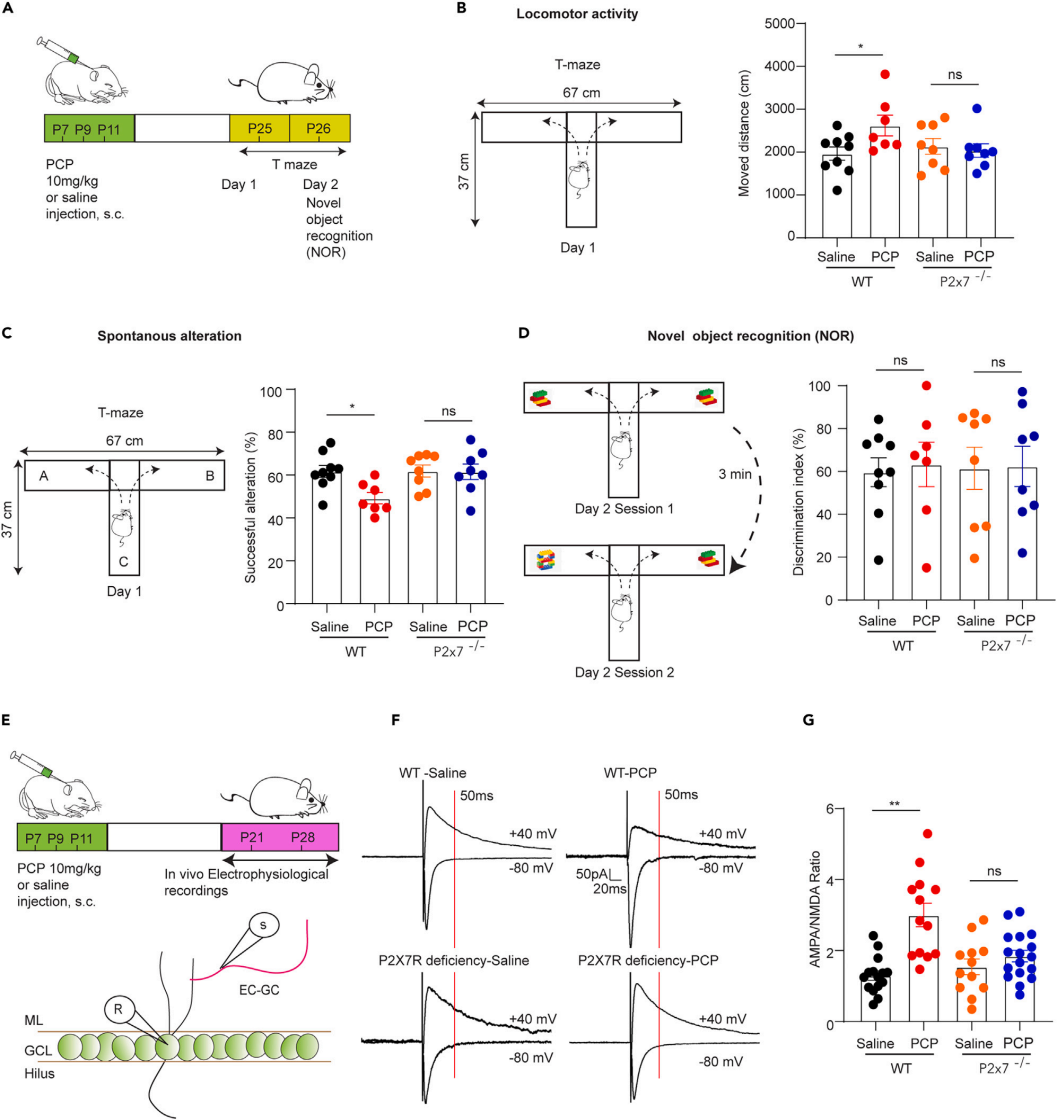
P2X7Rs deficiency alleviate schizophrenia-like behavior and restore EC-GC synapse alteration in juvenile (A) Schematic of the experiment. (B) Locomotor activity in T maze. PCP treated animals moved longer distance in comparison with saline treated group in WT, but both PCP and saline treated animals did not show any difference in P2X7R deficient groups (wt-saline: 1967 ± 155.8 cm, n = 9 vs. wt-PCP: 2623 ± 241.14 cm, n = 7, p = 0.047; P2x7^−/−^-saline: 2134 ± 183.6 cm, n = 8 vs. P2x7^−/−^-PCP: 2038 ± 158.5 cm, n = 8, p > 0.99, one-way ANOVA by Dunnett’s test). (C) Spontaneous alteration in T maze. PCP injection in WT showed less successful alteration compared the saline group (wt-saline: 61.71 ± 2.79%, n = 9 vs. wt-PCP: 49.20 ± 7.10%, n = 7, p = 0.02; P2x7^−/−^-saline: 61.88 ± 2.82%, n = 8 vs. P2x7^−/−^-PCP: 61.52 ± 3.59%, n = 8, p > 0.99, one-way ANOVA by Dunnett’s test). No change has been observed in the P2X7R deficient mice injected with PCP or saline. (D) The novel object recognition test. PCP treatment did not show any recognition preference (wt-saline: 59.61 ± 6.74%, n = 9 vs. wt-PCP: 63.25 ± 10.41%, n = 7, p > 0.99; P2x7^−/−^-saline: 61.47 ± 8.89%, n = 8 vs. P2x7^−/−^-PCP: 62.57 ± 9.40%, n = 8, p > 0.99, one-way ANOVA by Dunnett’s test). (E) The scheme for electrophysiological recordings. (F) The representative traces for AMPA/NMDA ratio under different conditions. (G) The AMPA/NMDA ratio in WT and P2X7R deficient mice treated with either PCP or saline. The AMPA/NMDA ratio was determined by calculating the AMPA current amplitude relative to NMDARs current amplitude at 50 m post-stimulus. PCP-treated mice showed a significantly higher AMPA/NMDA ratio than saline-treated mice in WT but not in P2X7R deficient (wt-saline: 1.31 ± 0.17, n = 12 vs. wt-PCP: 3.00 ± 0.33, n = 13, p < 0.0001; P2x7^−/−^-saline: 1.62 ± 0.25, n = 10 vs. P2x7^−/−^-PCP: 1.85 ± 0.16, n = 17, p = 0.95, one-way ANOVA by Dunnett’s test). The data are presented as the mean ± SEM. ∗ marks significant difference.

Next, we sought to determine whether P2X7Rs also participate in the regulation of pathological alterations of DG GCs excitatory neurotransmission. To this end, we injected the NMDA receptor selective antagonist PCP or vehicle (saline) into WT and P2X7R deficient male pups at P7, P9, and P11 to establish a neurodevelopmental model of SZ^30^^,^^31^ and performed a series of behavioral tests after weaning (Figure 6A). Intriguingly, we found that PCP treatment resulted in hyperlocomotor activity in WT mice but not in P2X7R deficient mice, indicating that the genetic ablation of P2X7Rs alleviated PCP-induced positive symptoms (Figure 6B). Although PCP did not change the recognition memory as the novel object recognition test did not show any difference across four groups (Figure 6D), we still found that PCP treated animals decreased the successful spontaneous alterations in T-maze in WT mice but not in P2X7R deficient mice (Figure 6C), suggesting that the genetic absence of P2X7R also reversed the working memory impairment. To further address the underlying mechanism, we next investigated whether EC-GC synapses alteration play a potential role. We measured the AMPA/NMDA ratio at EC-GC synapses (Figure 6E) on P21-28 after postnatal PCP or saline treatment and found that PCP treatment elevated the AMPA/NMDA ratio mainly by directly inhibiting NMDA components instead of altering AMPA components in WT mice (Figures 6F and 6G). Importantly, P2X7R deficient seemed to rescue NMDA receptor function, further contributing to recovery of the AMPA/NMDA ratio (Figures 6G, S4A, and S4B). Taken together, the results indicate that genetic deletion of P2X7Rs alleviates PCP-induced behavioral alterations in juvenile mice, by compensating for the function of NMDA receptors.

**Figure 7:**
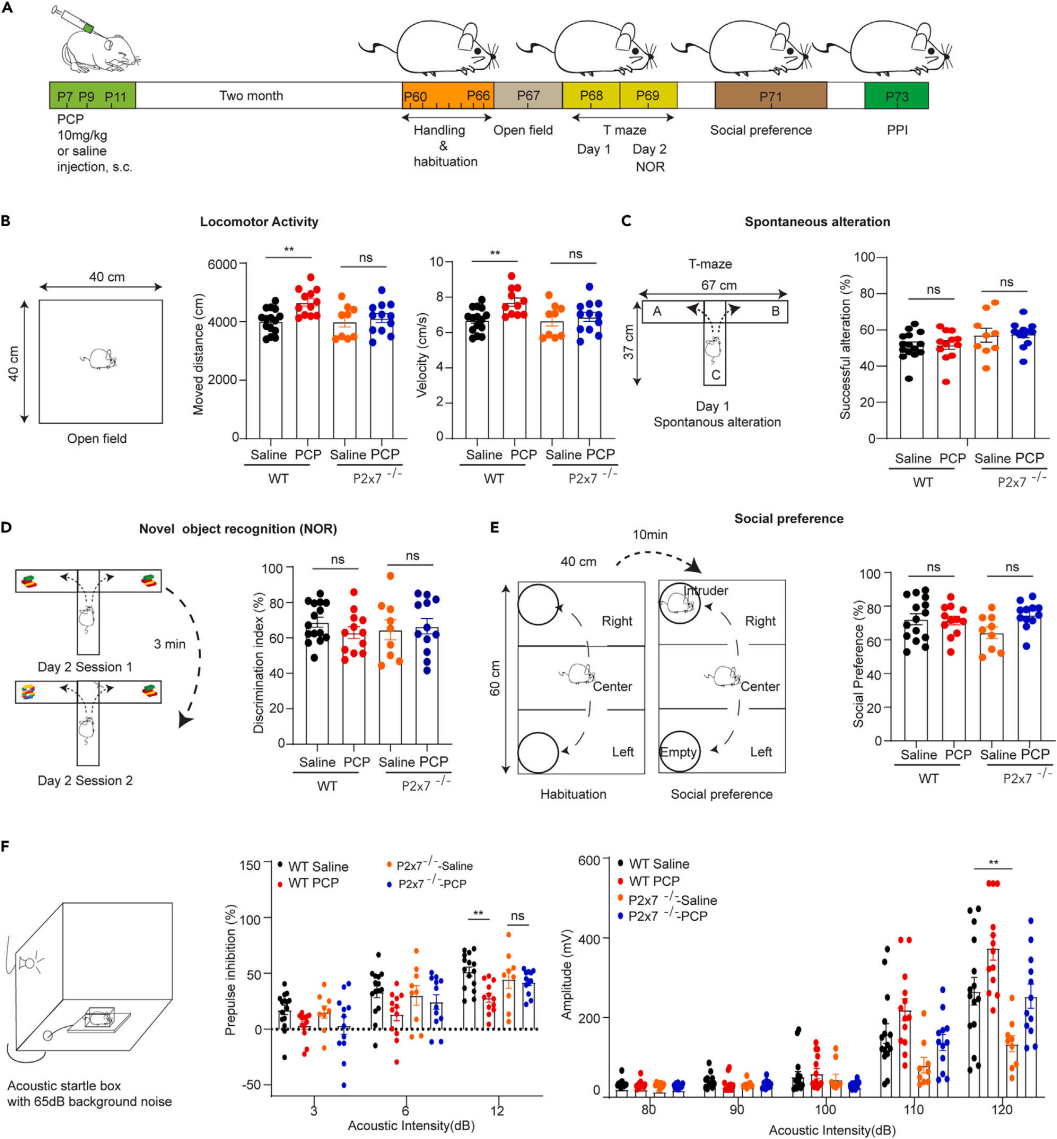
P2X7Rs deficiency alleviates schizophrenia-like behavior in adult (A) Scheme of the experiment. (B) Locomotor activity in the open field test. PCP-treated mice traveled a longer distance than saline-treated mice. However, the locomotor hyperactivity induced by PCP was significantly alleviated in the P2X7R deficient group (wt-saline: 4024 ± 106.70 cm, n = 15 vs. wt-PCP: 4662.50 ± 129.22 cm, n = 12, p = 0.035; P2x7^−/−^-saline: 4007.68 ± 185.78 cm, n = 9 vs. P2x7^−/−^-PCP: 4126.31 ± 149.16 cm, n = 12, p = 0.99, one-way ANOVA by Dunnett’s test). (C) Spontaneous activity test in T maze. PCP treatment group did not show a significant difference in both tests (wt-saline: 51.47 ± 1.99%, n = 15 vs. wt-PCP: 51.66 ± 2.41%, n = 12, p = 0.81; P2x7^−/−^-saline: 57.04 ± 3.88%, n = 9 vs. P2x7^−/−^-PCP: 57.69 ± 1.97%, n = 12, p = 0.79, one-way ANOVA by Dunnett’s test). (D) The novel object recognition in T maze. No difference was observed in different groups (wt-saline: 68.84 ± 2.81%, n = 15 vs. wt-PCP: 62.96 ± 3.34%, n = 12, p = 0.81; P2x7^−/−^-saline: 65.66 ± 6.25%, n = 9 vs. P2x7^−/−^-PCP: 66.56 ± 4.38%, n = 12, p = 0.79, one-way ANOVA by Dunnett’s test). (E) The scheme of the social preference test and no difference has been observed from 4 groups (wt-saline: 72.30 ± 3.22%, n = 15 vs. wt-PCP: 71.53 ± 2.61%, n = 12, p = 0.89; P2x7^−/−^-saline: 64.27 ± 3.47%, n = 9 vs. P2x7^−/−^-PCP: 74.91 ± 2.24%, n = 12, p = 0.13, one-way ANOVA by Dunnett’s test). (F) The acoustic startle setup. (F, middle) The PCP-treated group displayed a decrease in PPI, which was restored in the P2X7R deficient group (wt-saline: 51.55 ± 4.07%, n = 15, vs. wt-PCP: 28.09 ± 3.95%, n = 12, p = 0.001; P2x7^−/−^-saline: 44.69 ± 8.19%, n = 9 vs. P2x7^−/−^-PCP: 42.20 ± 3.12%, n = 12, p = 0.99, two-way ANOVA by Dunnett’s test). (F, right) The startle reflex in response to 80, 90, 100, 110, and 120 dB. Compared to WT, P2X7R deficient mice showed less response to 120 dB (P120: wt-saline: 266.27 ± 14,60 mV, n = 15 vs. P2x7^−/−^-saline: 134.39 ± 20.44 mV, n = 9, p = 0.009, two-way ANOVA by Dunnett’s test). The data are presented as the mean ± SEM. ∗ marks significant difference.

Next, we wondered whether genetic ablation of P2X7Rs also could resist the behavior alterations induced by postnatal PCP injection during adulthood (Figure 7A). We then examined a series of behavior paradigms at adult mice. Similarly, we observed the PCP-induced hyperlocomotor activity in WT but not in P2X7R deficient mice (Figure 7B). Unlike juvenile mice, we did not observe any difference in spontaneous alternations (Figure 7C) or novel object recognition in the T-maze (Figure 7D), suggesting that postnatal PCP-induced working memory impairment was probably restored by this age. No change has been found in the three-chamber social preference test that demonstrated that PCP did not affect social ability using this paradigm (Figure 7E). Compelling clinical studies have revealed that prepulse inhibition (PPI) impairment is an important hallmark of SZ,^32^^,^^33^^,^^34^^,^^35^ although this symptom is not observed in all animal models.^36^^,^^37^ Therefore, we examined the acoustic startle reflex in our postnatal PCP model. We found that PPI was significantly impaired, especially when we applied a 12 dB stimulus prior to a 120 dB stimulus, in WT mice treated with PCP postnatally compared to their saline-treated counterparts (Figure 7F, middle). In contrast to WT mice, P2X7R-deficient mice treated with PCP did not exhibit PPI impairment (Figure 7F, middle). Moreover, we found that P2X7R deficient mice showed less response to higher acoustic stimulus (Figure 7F, right). Meanwhile, we did not observe difference in interstimulus interval in startle reflex (Figure S4D; Table S3) and body weights were unaffected in response to PCP treatment (Figure S4C; Table S4).

Overall, the data demonstrated that the alleviation of PCP-induced SZ-like behaviors in the absence of P2X7R is probably mediated via a direct interaction with NMDA receptors.

## Discussion

The primary objective of this study was to examine whether functional P2X7Rs modulate excitatory neurotransmission of GC cells of the DG with a presynaptic mode of action. Here we report for the first time that activation of P2X7Rs regulate neurotransmission at this synapse both in AP-dependent and independent manner, input specifically, involving the pathway originating from the entorhinal cortex and P2X7R activation directly elicits Ca^2+^ influx from the boutons of the same pathway. Moreover, P2X7Rs ablation restored AMPA/NMDA ratio in EC-GC pathway after postnatal PCP injection, suggesting the potential mechanism for later onset of SZ-like behaviors.

### P2X7Rs regulate neurotransmission from presynaptic sites

As a first step, we showed that, the frequency, but not amplitude of both AMPA- and NMDA receptor-mediated sEPSCs and mEPSCs were decreased in the P2X7Rs deficient animals suggesting that in the WT mice synaptic transmission is subject to regulation by endogenous P2X7R activation with a presynaptic mode of action. Then we attempted to replicate this phenomenon with exogenous agonist application.

In whole hippocampal slices, activation of P2X7Rs by the agonist BzATP enhanced the release of neurotransmitters.^8^^,^^9^ Further patch-clamp experiments also demonstrated that P2X7R activation increases excitatory neurotransmission in hilar neurons^10^ but decreases in CA3 neurons.^38^ Later experiments showed that the suppressive effect of BzATP was attributed to the rapid breakdown of BzATP into adenosine, leading to inhibition caused by further activation of presynaptic A1 receptors.^39^ To minimize the enzymatic catabolism of BzATP, we puffed BzATP directly to the molecular layer of the DG and pre-perfused the brain slices with the NTPDase inhibitor, ARL-67156. ARL-67156 alone did not affect synaptic transmission in our system (Figure S2B). Although the increase in frequency induced by BzATP was abolished by bath perfusion of P2X7R selective antagonists JNJ-47965567 and A 438079, high concentration of BzATP might also activate other members of the P2X family^40^ as well as the metabotropic P2Y2Rs.^41^^,^^42^^,^^43^ Among other P2X receptors, at first we have focused on P2X4 receptors based on the data that P2X7R could form the heteromer with P2X4R^23^^,^^24^ and their expression can be regulated by each other.^44^ Moreover, P2X4 receptors have been found to participate in the modulation of synaptic transmission and plasticity in the hippocampus.^20^^,^^45^^,^^46^ We found that the expression of P2X4 was robustly upregulated in P2X7Rs deficient mice compared to WT mice (Figure 2G). Moreover, the selective P2X4Rs antagonist 5-BDBD application blocked BzATP effect in P2X7Rs deficient mice (Figure 2H), ruling out the participation of other subtypes than P2X7 and P2X4. Of note, P2X7R mouse model used in this paper is not a full knockout, as it also expresses functional splices variants that escape from the gene inactivation strategy.^47^^,^^48^

It is well-known that extracellular calcium in physiological concentrations acts as a negative allosteric modulator of P2X7R by reducing the affinity of receptors for orthosteric ligand agonists.^49^^,^^50^ Thus, although a low Ca^2+^ (0.5 mM)/0 Mg^2+^ ACSF is routinely used to investigate the electrophysiological role of P2X7Rs in brain slices,^11^^,^^51^ the physiological calcium concentration is typically around 1.3 mM and varies from 1.3 mM to 1.8 mM (normally below 1.8 mM) depending on arousal/sleep or other situations.^52^^,^^53^^,^^54^ Therefore, in our study, we utilized both 1.3 mM Ca^2+^/2 mM Mg^2+^ containing physiological ACSF and low Ca^2+^ (0.5 mM)/0 Mg^2+^ ACSF and found that BzATP was able to induce P2X7R-mediated response in both cases. In contrast, when we elevated calcium in ACSF to 2.6 mM, it completely abolished BzATP-induced effect, supporting the involvement of P2X7R in its action.

A recent study reported that the BzATP-induced increase in sEPSCs frequency can be abolished by inhibiting the metabolism of astrocytes with the glial metabolic blocker FAC in the CA1 and CA3 regions, but not in the DG, of the hippocampus.^11^ Due to the nonspecific nature of long-term FAC treatment,^55^ we did not utilize this approach in our experiments. Instead, we assumed that if astrocytic P2X7Rs regulated excitatory neurotransmission, it would not have any input specificity. To investigate this possibility, we extracellularly stimulated two major excitatory inputs to DG GCs, the EC-GC pathway and MC-GC pathway.^25^ We subsequently found that the regulatory effect of P2X7Rs was mainly derived from the EC-GC pathway but not the MC-GC pathway in both normal and low divalent cations conditions. Findings from P2X7 immunostaining and calcium imaging in axonal boutons further confirmed the direct involvement of neuronal P2X7Rs. However, the potential role of astrocytic P2X7Rs needs further investigation in future.

EC-GC pathway contains LPP and MPP paths. Although the two paths can be differentiated pharmacologically and electrophysiologically, we primarily employed paired-pulse protocols in which two consecutive stimuli were delivered with a 50 ms interstimulus interval (ISI). This conventional protocol was believed to induce paired-pulse facilitation (PPF) in the LPP and paired-pulse depression (PPD) in the MPP in the presence of 2 mM extracellular calcium,^56^^,^^57^^,^^58^^,^^59^ although a later study demonstrated that both pathways induce PPF of field excitatory postsynaptic potentials (fEPSPs) at an ISI of 50 ms.^60^ In our system, we found that the stimulated fibers from the LPP and MPP showed PPF when used 1.3 mM calcium-containing ACSF. However, the PPR value from MPP was still lower than from LPP. Importantly, LPP and MPP were also specifically differentiated by injected axon-target GCaMP6s into LEC and MEC. Therefore, the regulatory effect of P2X7Rs did not display significant difference in LPP and MPP.

In addition to electrophysiology to uncover potential P2X7R mediated postsynaptic changes, we have also quantified the number of dendritic spines in the DG region of the hippocampus. In fact, the findings of the present and our previous study^61^ altogether shows that only the pyramidal cells from CA1 but not from CA3 and DG region had the morphological deficit in P2X7R deficient mice compared the WT group, indicating the subregion-specificity of such alterations. Interestingly, Sebastian Serrano^62^ detected a reduction of dendritic spines is in P2X7R deficient mice at P9, whereas, the animals we included into this paper study were P21–28 days old. Therefore, the cell-type specificity and age might also influence the impact of P2X7R on morphological changes.

### Genetic deficiency of P2X7Rs alleviates PCP-induced schizophrenia (SZ)-like symptoms

SZ is a highly complex multifactorial human neurodevelopmental psychiatric disorder, which is due to interactions between genetic and environmental factors; therefore, it cannot be replicated in a single model in rodents. The compelling evidence showing that NMDARs antagonist PCP administration resulted in SZ-like symptoms has been primarily collected in support of the glutamate hypothesis of schizophrenia psychopathology.^12^ Nevertheless, there are a number of studies showing the successful reproduction of SZ relevant symptoms induced by the NMDAR hypofunction model, including positive,^14^^,^^15^^,^^17^^,^^63^ negative,^64^ and cognitive symptoms.^17^^,^^63^ In our NMDAR hypofunction SZ model, we also successfully reproduced the hyperlocomotor activity and working memory deficit. The deficits in central inhibitory mechanisms have been found in patients with SZ by testing the startle response, especially prepulse inhibition. Prepulse inhibition is neurological phenomenon and refers to a weaker prestimulus (prepulse) could inhibit the startle response to a subsequent strong stimulus (pulse) via a central inhibitory or filtering mechanism. Therefore, prepulse inhibition reduction has been commonly found in SZ patients, even has been regarded as a hallmark/biomarker of SZ.^65^^,^^66^^,^^67^^,^^68^^,^^69^ In rodents, PPI deficit induced by PCP also has been documented.^70^^,^^71^^,^^72^^,^^73^ We also observed PPI deficit after postnatal NMDAR hypofunction induced by PCP injection.

Now there is growing evidence on the participation of P2X7Rs in the pathophysiology of SZ. Recently, the largest GWAS study done ever on schizophrenic subjects identified gene polymorphisms of P2RX7 associated with the disorder.^13^ In rodents, a number of studies showed the potential role of P2X7R in SZ-like symptoms, especially in NMDAR hypofunction SZ model. Pharmacological blockage and genetic deficiency of P2X7Rs alleviated PCP-induced SZ-symptoms, including hyperlocomotor activity, PPI impairment, and spatial memory deficit, in acute and subchonic models.^14^^,^^15^^,^^17^ Consistent with these studies, we also found P2X7R suppressed hyperlocomotion at both juveniles and adults, working memory deficit and sensory gating system impairment. Nevertheless, we should note that due to different construct mechanism, using the maternal immune activation model, a slightly different behavioral profile and its regulation by P2X7R was detected.^61^

It is now became widely accepted that environmental influences, i.e., pre- or postnatal stressors, play a major role in the etiopathophysiology of SZ, including the cognitive symptoms.^74^ Therefore, we assume that PCP treatment, as a postnatal stressor lead to elevated extracellular ATP level and overactivation of P2X7R in our experiments, which contribute to behavioral alterations, subject to attenuation by P2rx7 gene deficiency (Figures 6 and 7). To support this assumption, we recently demonstrated the elevated serum ATP levels and extracellular ATP in schizophrenic patients in a clinical study^75^ and other experimental models of neurodevelopmental psychiatric disorders,^61^^,^^76^ respectively.

The underlying mechanism of action of P2X7Rs in alleviating PCP-induced behavioral alterations has been investigated in different ways. A previous study showed that fewer action potentials were generated in response to the same current injection in P2X7R deficient animals compared to WT animals and that the number of neurons activated by PCP was decreased in the medial prefrontal cortex (mPFC) pyramidal neurons in P2X7R-deficient animals.^15^ In our study, we did not find any difference in the membrane properties or intrinsic excitability of DG GCs between mice of the two genotypes, although there was an age-related change in amplitude of action potential. Therefore, the impact of P2X7Rs on neuronal activation might be different depending on brain regions and cell types. Other studies have focused on the interaction between NMDARs and P2X7Rs. By examining NMDA-induced current changes in prefrontal cortex (PFC) pyramidal neurons in the genetic absence and pharmacological blockade of P2X7Rs, we reported an interaction between P2X7Rs and NMDA receptors in pyramidal neurons.^14^ In our present experiments, we found PCP administration causes a higher AMPA/NMDA ratio mainly by blocking NMDA components and suppression of NMDA components is rescued by genetic ablation of P2X7Rs. Therefore, it is plausible that genetic deficiency of P2X7Rs alters the susceptibility of postsynaptic NMDARs to PCP right after the treatment. In fact, genetic deletion of P2X7Rs has been reported to alter NMDA receptor subunits during the process of neurodevelopment. For example, increased mRNA expression of Grin2b in the PFC in young adult P2X7R deficient mice and increased mRNA expression of Grin2a in the hippocampus in juvenile P2X7R deficient mice.^14^ However, the exact mechanism of this pathological interaction between P2X7Rs and NMDA receptors requires further investigation.

### Limitations of the study

The potential glial contribution to the P2X7R mediated modulation revealed by the study is not excluded. It requires further investigation to address what types of mechanisms are involved in the compensation of NMDA receptors components of PCP-induced EC-GC synapse alteration restored by genetic deficiency of P2X7Rs to alleviate the schizophrenia-like symptoms.

## STAR★Methods

### Key resources table

**Table:** 

REAGENT or RESOURCE	SOURCE	IDENTIFIER
**Antibodies**		

Rabbit anti-P2X7	Alomone Labs	Cat. #.APR-004; RRID: AB_2040068
Rabbit anti-P2X4	Alomone Labs	Cat. #. APR 002 RRID: AB_26628-22-8
Rabbit anti-β-actin	Cell Signaling Technology	Cat. #. 4967 RRID:AB_330288
Chicken anti-GFP	Aves Labs	Cat. #. GFP-1020 RRID: AB_10000240
488 goat anti-chicken IgY	Invitrogen	Cat. #. A11039; RRID: AB_2534096
Streptavidin, Alexa Fluor™ 594 conjugate	Invitrogen	Cat. #. S11227; RRID:AB_2313574
Rabbit anti-Homer1	Synaptic System	Cat. #. 160 003 RRID: AB_887730
Guinea pig Anti-Vesicular Glutamate Transporter 1 (VGlut1) Polyclonal antibody, Unconjugated	Millipore	Cat. #. AB5905 RRID:AB_2301751
Chicken anti-GFP	Biozol	Cat. #.GFP-1020; RRID:AB_10000240
Cy™3 AffiniPure Donkey Anti-Guinea Pig IgG (H + L)	Jackson Immuno Research	Cat. #. 706-165-148 RRID: AB_2340460
Alexa Fluor® 647 AffiniPure Donkey Anti-Rabbit IgG (H + L)	Jackson Immuno Research	Cat. #. 711-605-152 RRID: AB_2492288
Alexa Fluor® 488 AffiniPure Donkey Anti-Chicken IgY (IgG) (H + L)	Jackson Immuno Research	Cat. #. 703-545-155 RRID: AB_2340375

**Bacterial and virus strains**		

pAAV-hSynapsin1-axon-GCaMP6s	Addgene	Addgene:111262

**Chemicals, peptides, and recombinant proteins**		

DL-AP5	Tocris Bioscience	Cat. #.0105/10; CAS. #: 76326-31-3
SR 95531 hydrobromide	Tocris Bioscience	Cat. #.1262/10; CAS. #: 104104-50-9
CNQX disodium salt	Tocris Bioscience	Cat. #.1045/1: CAS. #:479347-85-8
ARL 67156 trisodium salt	Tocris Bioscience	Cat. #.1283/10: CAS. #:1021868-83-6
QX 314 chloride	Tocris Bioscience	Cat. #.2313/50: CAS. #: 5369-03-9
JNJ 47965567	Tocris Bioscience	Cat. #.5299/10: CAS. #:1428327-31-4
A 438079 hydrochloride	Tocris Bioscience	Cat. #. 2972; CAS.#. 899431-18-6
5-BDBD	Tocris Bioscience	Cat.#. 3579 CAS. #.768404-03-1
Hoechst 33342	Tocris Bioscience	Cat. #.5117/50; CAS. #:875756-97-1
Tetrodotoxin citrate free	Alomone Labs	Cat #: T-560; CAS No.: 13072-89-4
ω-agatoxin TK	Alomone Labs	Cat #: STA-530; CAS #.: 158484-42-5
ω-conotoxin GVIA	Alomone Labs	Cat #: C-300; CAS #.: 106375-28-4
2′(3′)-*O*-(4-Benzoylbenzoyl)adenosine 5′-triphosphate triethylammonium salt	Sigma-Aldrich	Cat. #. B6396 CAS #.:112898-15-4
DCG IV	Tocris Bioscience	Cat. #. 0975; CAS No.: 147782-19-2
Biocytin	Biotum	Catalog #: 90060
D-serine	Tocris Bioscience	Cat. #. 0226; CAS. #.: 312-84-5

**Deposited data**		

Original western blot image	Mendeley	https://doi.org/10.17632/swxsk5mgxb.1

**Experimental models: Organisms/strains**		

C57BL/6J	The Jackson Laboratory	Cat. #.UMB1455
P2rx7−/− breeding pairs [14]	Pfizer, Inc Dr. Christopher Gabel	N/A

**Software and algorithms**		

Suite2P	GitHub	version 0.8.0, RRID: SCR_016434; https://www.github.com/MouseLand/suite2p
Clampfit 10.4	Molecular Devices	https://www.moleculardevices.com/
Python spyder	The spyder Development Team	https://www.spyder-ide.org/
Anaconda	Anaconda Inc	https://www.anaconda.com/
NIS element	Nikon	https://www.microscope.healthcare.nikon.com/products/software/nis-elements
Huygen	Huygens computer engine	https://svi.nl/Huygens-Deconvolution
GraphPad Prism	GraphPad Software Inc.	GraphPad Prism 8.0.2

### Resource availability

#### Lead contact

Further information and requests for resources should be directed and will be fulfilled by the lead contact, Beata Sperlagh (sperlagh@koki.hu)

#### Materials availability

This research project did not generate new unique reagents and techniques.

### Experimental model and study participant details

#### Animal models

For all the experiments, wt and P2X7R deficient mice on the C57Bl/6J background were used bred in the local animal house. The original breeding pairs of P2X7R deficient mice were provided by Dr. Christopher Gabel (Pfizer, Inc.; Groton, CT, USA). The P2x7^−/−^ mice harbored P2X7-F1 (5′-CGGCGTGCGTTTTGACATCCT-3′) and P2X7-R2 (5′-AGGGCCCTGCGGTTCTC-3′).^14^ Postnatal day (P)21–P28 and P58-P69 C57Bl/6J mice of either sex were used for the electrophysiological experiments. For virus injection, P40-P47 home-bred male C57Bl/6J mice were used. After 2 weeks of virus expression, the mice were sacrificed at P54 to P61 for calcium imaging experiments. To establish a PCP-induced SZ model, male mice aged 13 weeks old and female mice aged 13 weeks old on the C57Bl/6J background were mated. PCP-HCl from Lipomed (Lot. 436.1B3.2) was freshly dissolved in saline for each batch of mice. PCP (10 mg/kg) or an equal volume of saline was subcutaneously injected into the dorsal region of male mice on P7, P9, and P11 between 10:00 and 11:00 a.m. All male pups from the same litter received the same treatment. PCP- or saline-injected mice were weaned at 3 weeks of age, and 3–5 animals were housed in each cage.At P25-26, male mice were utilized for T-maze experiments. After 2 months, P67 male mice were utilized for a series of behavioral tests. All animals were housed on a standard 12 h light/12 h dark cycle with *ad libitum* access to food and water. Because SZ is more frequent in males than females, in behavior experiments we primarily used male mice. However, because the course of SZ may differ between the two sexes, it is essential to investigate the participation of P2X7R in SZ models also in female mice. Animal handling, experimental protocols and sacrifice procedure followed a protocol approved by the Animal Welfare Committee of the Institute of Experimental Medicine and by the respective authorities (Budapest, Hungary, ref. No. PEI/001/778–6/2015, PE/EA/297-1/2021) in accordance with the European Communities Council Directive of 24 November 1986 (86/609/EEC). This research complies with all the guidelines of our institution’s animal welfare committee and this journal’s ethics policies.

### Method details

#### Acute brain slice preparation

Hippocampal brain slices were obtained from P21 to P28 and P58 to P69 wt and P2X7R deficient mice on the C57Bl/6J background. Mice of either sex were first anesthetized by inhalation of forane and decapitated. The brains were quickly removed and cut into 300 μm slices using a vibratome (Leica VT1200, Germany) filled with ice-cold cutting solution (in mM: 85 NaCl, 2.5 KCl, 0.5 CaCl_2_, 1.25 NaH_2_PO_4_, 24 NaHCO_3_, 25 glucose and 75 sucrose; pH 7.4, 290–300 mOsm) bubbled with 95% O_2_ + 5% carbon dioxide (CO_2_). After 30 min of incubation at 34°C, the slices were stored at room temperature in ACSF (in mM: 126 NaCl, 2.5 KCl, 2 CaCl_2_, 2 MgCl_2_, 1.25 NaH_2_PO_4_, 26 NaHCO_3_ and 10 glucose; pH 7.4, 300–310 mOsm) bubbled with 95% O_2_ + 5% CO_2_.

#### Whole-cell patch-clamp recordings

All recordings were performed at room temperature. Hippocampal slices were submerged in a chamber and perfused with oxygenated ACSF at a rate of 2.5 mL/min. Previous studies have reported that calcium in physiological concentrations acts as a negative allosteric modulator of P2X7R^11^^,^^49^ and NMDA receptors are blocked by Mg^2+^ in a voltage dependent way. Therefore, we created a low divalent cation (X2+) solution, Mg^2+^ was omitted from the ACSF, and the Ca^2+^ concentration was decreased to 0.5 mM. However, normal ACSF containing 1.3 mM Ca^2+^/2 mM Mg^2+^ has also been used in input-specificity experiments and calcium imaging experiments. Whole-cell patch-clamp experiments were performed using an Axopatch 1D amplifier and Digidata 1322A data acquisition system (Molecular Devices, California, US). DG GCs were visualized using differential interference contrast (DIC) microscopy with an Olympus BX50WI microscope and a 40x water immersion objective (Lumpleln 40xWI, 0.8 NA, Olympus, Hamburg, Germany). Borosilicate glass pipettes (Harvard Apparatus, Massachusetts, UK) with a resistance of 4–7 MΩ were pulled with a Sutter P-2000 micropipette puller (Sutter Instruments, Novato, California, US).

For the voltage-clamp model, signals were filtered at 2 kHz and digitized at 10 kHz. AMPA- and NMDA receptor-mediated excitatory postsynaptic currents (EPSCs) were obtained at a holding potential of −80 mV using a patch pipette filled with an intracellular solution of the following composition (in mM): 120 CsCl, 8 NaCl, 10 HEPES, 0.3 Na-GTP, 4 Mg-ATP, 10 PO creatine, and 8 biocytin, pH 7.2, 290–300 mOsm). To record AMPA receptor-mediated sEPSCs, the GABA_A_ receptor antagonist SR 95531 (10 μM) and NMDA receptor antagonist DL-AP5 (50 μM) were added to the ACSF, while the sodium channel blocker TTX (1 μM) was added to ACSF containing SR 95531 and DL-AP5 to record AMPA receptor-mediated mEPSCs. To record NMDA receptor-mediated sEPSCs, SR 95531 (10 μM), the AMPA receptor antagonist CNQX (20 μM) and NMDA receptor co-agonist D-serine (50 μM) were added to 0.5 mM Ca^2+^/0 mM Mg^2+^ACSF. TTX (1 μM) was added to 0.5 mM Ca^2+^/0 mM Mg^2+^ACSF containing SR 95531 (10 μM), D-serine (50 μM) and CNQX (20 μM) to record NMDA receptor-mediated mEPSCs. Furthermore, a sodium channel blocker QX314 (5 mM) was added to the internal solution to record AMPA- and NMDA receptor-mediated mEPSCs. The series resistance was continuously monitored using 100-ms-long −5 mV voltage steps.

Evoked EPSCs were generated with a stimulation isolation unit (World Precision Instruments, Sarasota, FL, US). A bipolar electrode (World Precision Instruments, Sarasota, FL, US) was placed either in the perforant path or the outer border of the GC layer ∼100 μm rostral to the recording electrode. The internal solution contained (in mM: 115 Cs-methanesulfonate, 8 NaCl, 10 HEPES, 0.3 Na-GTP, 4 Mg-ATP, 10 PO creatine, 8 biocytin, and 5 QX314, pH 7.2, 280–290 mOsm). SR 95531 (10 μM) was added to the ACSF to continuously block GABA_A_ receptors. For PPR recording, a paired-pulse protocol involving application of two stimuli at an interpulse interval of 50 m was used, and the cells were voltage-clamped at −80 mV. The PPR was calculated as the second peak amplitude (P2) divided by the first peak amplitude (P1). To determine the AMPA/NMDA ratio, currents evoked at −80 mV were identified as AMPAR-mediated currents. The NMDA component amplitude was determined 50 ms after the peak at +40 mV, when the AMPAR component was abolished.^77^^,^^78^^,^^79^^,^^80^ The AMPA/NMDA ratio was calculated as the AMPA current amplitude relative to the NMDA current amplitude at 50 m poststimulus (average of 10–15 sweeps).

For the current-clamp model, electrodes were filled with solution composed of (in mM: 126 K-gluconate, 4 KCl, 10 HEPES, 0.3 Na-GTP, 4 Mg-ATP, 10 PO creatine, and 8 biocytin, pH 7.2, osmolarity between 290 and 300 mOsm). To elicit APs, somatic currents were injected from −100 pA to +280 pA for 450 ms with an increment of 20 pA. Data acquisition was performed using pCLAMP 10.2 software (Molecular Devices, California, US). Several membrane parameters, including 1) the input resistance (IR), which was determined from the first current step at −100 pA; 2) the maximal frequency, at 280 pA; and 3) the action potential (AP) amplitude (from the threshold), rise time, half-width time and afterhyperpolarization (AHP) amplitude, which were determined manually after data differentiation for the first AP. Cells were excluded if the holding current was larger than −100 pA.

#### Western blot

Experiments were performed on young (21–28 days) littermate control or P2X7R deficient mice. Experimental animals were euthanized with gradual filling of CO_2_ inhalation, decapitated and the hippocampi were dissected on ice and were snap frozen in liquid nitrogen. Tissue samples were lysed in radioimmunoprecipitation assay (RIPA) buffer containing 150 mM NaCl, 50 mM Tris-HCl (pH 7.4), 5 mM EDTA, 0.1% (w/v) SDS, 0.5% sodium deoxycholate and 1% Triton X-100 as well as protease inhibitors (10 mg/mL leupeptin, pepstatin A, 4-(2-aminoethyl) benzensulfonyl-fluorid and aprotinin). Total lysates were separated by sodium dodecyl sulfate-polyacrylamide gel electrophoresis. Protein was then transferred onto nitrocellulose membranes, unspecific binding was prevented by blocking in 5% non-fat dry milk for 1h at room temperature (RT) and followed by overnight incubation with primary antibodies (anti-P2X4R antibody, 1:200, Alomone Labs; anti-β-actin, 1:1000, Cell Signaling Technology Inc.). Membranes were incubated with horseradish peroxidase-conjugated secondary antibodies (Cell Signaling Technology Inc., Danvers, MA, USA) for 1 h at RT and were developed using the ECL detection system (Thermo Scientific Pierce, Life Technologies). Protein band intensities were analyzed by ImageJ software (NIH). Intensity values of bands representing P2X4R proteins (50–55 kDa) were normalized to the intensity of the band representing total protein (β-actin: 42–45 kDa).

#### Immunostaining of biocytin-labeled DG GCs

After each recording, the recording pipette was quickly retracted from the neuron to help maintain cell integrity for histology. The slices were then fixed overnight with 4% paraformaldehyde (PFA) at 4°C. On the following day, the slices were washed with 0.1 M phosphate-buffered saline (PBS) 3 times for 10 min each, followed by 0.05% Tris-buffered saline (TBS) 3 times for 10 min each, and then blocked with 5% normal goat serum (Vector Laboratories Inc., Burlingame, California, US) containing 0.03% Triton X-100 for 30 min. The slices were subsequently incubated in Alexa Fluor 594-conjugated streptavidin (Thermo Fisher Scientific, Massachusetts, US) diluted 1:500 in 0.03% Triton X-100 for at least 4 h. Then, we repeatedly washed the slices with TBS and PBS and mounted them on gelatin-coated glass slides using Vectashield mounting medium without DAPI (Vector Laboratories Inc., Burlingame, California, US). The examined cells were morphologically identified based on confocal images (Nikon C2 Nikon Europe, Amsterdam, The Netherlands).

#### Morphology and spine density quantification of biocytin-filled DG GCs

z stack confocal images used to analyze dendritic outgrowth were imported into Neurolucida software (MBF Bioscience, Williston, VT, US). The number of intersections was determined every 2 μm at a radius of 5 μm from the center of the neuronal soma. To visualize dendritic spines, 300 μm thick biocytin-labeled slices were removed from the slide and stored in 0.1 mM PB. After 3 washes for 10 min each with 0.1 mM PB, the slices were embedded in 2% agarose gel and further cut into 50 μm sections with a vibratome (Leica VT1200s, Germany). Then, the 50 μm slices were remounted on the slide with Fluoroshield for visualization with a Nikon C2 confocal microscope. A 60x oil immersion objective (NA 1.4) was used. Z-stacks were acquired at an interval of 0.125 μm, 0.08 pixel/um. The dendritic branches were divided into segments 100, 150, and 200 μm away from the DG GC soma. The original images were further deconvoluted by using Huygens Professional version 19.04 (Scientific Volume Imaging, The Netherlands, http://svi.nl) before quantification and then imported into Neurolucida software (Micro Bright Field, VT, US) to trace the spines and quantify the number of spines every 30 μm.

#### Virus injection and expression

pAAV-hSynapsin1-axon-GCaMP6s was a gift from Lin Tian (Addgene plasmid # 111262; http://n2t.net/addgene:111262; RRID:Addgene_111262), and was aliquoted and stored at −80°C before use. P40-47 male mice were anesthetized with ketamine/xylazine-hydrochloride (10 mL/kg body weight, i.p.) and then placed in a stereotactic frame. After removal of the skin, a small craniotomy was made above the EC (LEC: −3.8 mm anteroposterior (AP), ±3.9 mm mediolateral (ML) and −4.7 mm dorsal-ventral (DV); MEC: −4.72 mm (AP), ±3 mm (ML), −4 mm (DV)). The virus was diluted 1:10 with sterilized 0.1 mM PB (for the expression experiment, the same volume of purified virus was used). Sixty nanoliter of virus solution was slowly delivered with a glass capillary connected to a MicroSyringe Pump Controller (Nanoliter Injector 2010W, World Precision Instruments, Sarasota, FL, US) at a rate of 57 nL/s for 20 nL, then 100 nL/min for 40 nL. Following injection, the capillary was kept in place for 5 min before retraction. The scalp incision was closed with surgical sutures (Dafilon, Braun, Spain). After recovery from anesthesia on a heating pad, the mice were returned to their home cages. Postinjection analgesia was provided for 2 days to aid recovery. After 2 weeks, virus expression experiments were performed.

After 2 weeks, the virus-infected mice were anesthetized with gradual CO_2_ inhalation and then transcardially perfused with 0.9% NaCl (20 mL) for 5 min, followed by 4% PFA (50 mL) for 20 min. The brain was dissected out, further postfixed with 4% PFA overnight, and then stored in 0.1 mM PB at 4°C. The fixed brains were cut into 50 μm thick coronal and transverse sections with a vibratome (Leica VT1200s, Germany) and subsequently incubated with Hoechst 33342 (1:5000) (Tocris Bioscience, Avon, UK) for 10 min. The slices were further washed once for 10 min with 0.1 mM PB and then mounted on gelatin-coated glass slides using VectaShield without DAPI (Vector Laboratories Inc., Burlingame, California, US) overnight. Images were taken using a Nikon C2 confocal microscope (Amsterdam, The Netherlands) with 4x, 20x, and 60x objectives.

#### Immunostaining for virus injected animals

Virus-injected (1:10) wt and P2X7R deficient animals were perfused transcardially with 0.9% HCl, followed by 4% PFA. The brains were postfixed with 4% PFA overnight and then sliced into 40 μm sections with a Leica vibratome (Leica VT1200s, Germany). The brain sections were first incubated in citrate buffer (pH 6.0) for 30 min at 85°C and then washed 3x with 0.01 M PBS prior to blocking. The brain slices were incubated in blocking buffer containing 1% BSA, 5% FBS and 0.2% Triton diluted in 0.01 M PBS for 1 h at RT. A rabbit anti-P2X7R primary antibody (APR-004, Alomone Labs, Israel) was diluted 1:100 in blocking buffer, and a chicken anti-GFP primary antibody (GFP-1020, Aves Labs, CA) was diluted 1:1000 in blocking buffer. The slices were incubated in primary antibody on a shaker for two nights at 4°C. After washing with 0.01 M PBS 3 times, the sections were further incubated with biotinylated antibody solution (Vectastain kit, Vector Laboratories Inc., Burlingame, California, US) containing 10% BSA, goat serum, and biotinylated anti-rabbit IgG for 1 h at RT. Then, Alexa Fluor 594-conjugated streptavidin secondary antibody (Thermo Fisher Scientific, Massachusetts, US) was diluted 1:500 in blocking buffer, and Alexa Fluor 488-conjugated anti-chicken antibody (Thermo Fisher Scientific, Massachusetts, US) was diluted 1:1000. The slices were incubated in secondary antibody for 1 h at RT. Subsequently, the sections were mounted on coverslips with ProLong Gold Antifade (Thermo Fisher Scientific, Massachusetts, US) for visualization with a C2 confocal microscope. z stack images obtained at an interval of 0.125 μm, 0.08 pixel/um with a 60x objective were used for deconvolution with Huygen professional software (Huygens computer engine 4.2.1p764b). Then, the deconvoluted images were further imported into NIS-elements software (Nikon Instruments Inc. US) for XYZ dimension identification. We only quantified the number of P2X7Rs puncta localized on bouton per 50 boutons in one picture.

For presynaptic marker VGlut1 and postsynaptic marker Homer1 immunostaining, the brain sections were washed in 0.1 M PB buffer, followed by 0.5 M TBS prior to blocking. The brain slices were further incubated in blocking buffer containing 10% normal horse serum (NHS) and 0.3% Triton diluted in 0.5 M TBS for 1 h at RT, and then incubated with primary antibodies at 4°C for at least 72 h. Primary antibodies used: Guinea pig Anti-Vglut1 (Millipore, AB_2301751,1:1000), Rabbit anti-Homer1 (Synaptic System, AB_887730, 1:500) and Chicken anti-GFP (Biozol, GFP-1020, 1:10000). Slices were further rinsed in 0.5 M TBS and incubated with secondary antibodies at 4°C for overnight. Secondary antibodies used: Cy3 AffiniPure Donkey Anti-Guinea Pig IgG (H + L) (Jackson Immuno Research, AB_2340460, 1:500), Alexa Fluor 647 AffiniPure Donkey Anti-Rabbit IgG (H + L) (Jackson Immuno Research, AB_2492288,1:500) and Alexa Fluor 488 AffiniPure Donkey Anti-Chicken IgY (IgG) (H + L) (Jackson Immuno Research, AB_2340375, 1:500). Sections were further washed in 0.5 M TBS, followed by 0.1 M PB. Then we mounted the slices on coverslips with ProLong Gold Antifade (Thermo Fisher Scientific, Massachusetts, US) for visualization with a C2 confocal microscope. z stack images obtained at an interval of 0.125 μm, 0.08 pixel/um with a 60x objective were used for deconvolution with Huygen professional software (Huygens computer engine 4.2.1p764b).

#### Calcium imaging

After 2 weeks of expression of pAAV1-hSynapsin1-axon-GCaMP6s, the mice were anesthetized by inhalation of forane and decapitated to extract the brain. Transverse slices (300 μm) were used (acute brain slices were prepared ass described previously). An upright A1R MP + multiphoton confocal microscope (Nikon, Amsterdam, The Netherlands) with a water immersion objective of NA 1.10, WD 2.0 (CFI75 Apochromat 25XC W 1300) and a 680–1040 nm titanium sapphire laser were utilized to view and scan the slices. Ca^2+^ imaging was carried out using a resonant scanner at a frequency of 30 Hz, wavelength of 920 nm, and laser power of 15 mW. Extracellular electrical pulses were delivered using a sharp electrode filled with ACSF solution via a monopolar stimulus isolator unit to trigger axon calcium transients (BioStim STE-7c, Supertech Instruments UK, Ltd., UK). Five pulses were delivered by brief depolarizing current injection at frequencies of 10–100 Hz (10, 30, 60, and 100 Hz) via a Multiclamp 700B amplifier (Molecular Devices, California, US). The scanning duration was 10 s, and 15 scans total were obtained. Scan images were captured with NIS-elements software (Amsterdam, The Netherlands) for further data analysis. Average NIS images of 15 scans for each plane were converted from nd format to tif format using an edited script in Spyder using Python (Pierre Raybaut, The Spyder Development Team, Python 3.8) on the Anaconda platform (Anaconda, Inc., version 3.8). The tif format files were subsequently imported into the most cited calcium imaging toolboxes suite2p (suite2p, version 0.8.0, RRID: SCR_016434) to extract fluorescence traces and spikes from the region of interest (ROIs) using the following equation:

F_c_= F-0.7∗F_neu_

where F_c_ is the corrected fluorescence value, F is the raw fluorescence intensity, F_neu_ is the neuropil-induced fluorescence intensity, and 0.7 is the neuropil coefficient. To further calculate the electrical pulse-induced fluorescence intensity, we used a script in Spyder using Python (Pierre Raybaut, The Spyder Development Team, Python 3.8) on the Anaconda platform (Anaconda, Inc., version 3.8) to subtract the baseline fluorescence intensity from the peak fluorescence intensity and obtain the change in fluorescence intensity, also known as the ΔF.

#### Behavioral tests

After PCP or saline injection, T maze spontaneous alteration and novel object recognition were tested at P25-P26. Two months after PCP or saline injection, mice were subjected to a series of behavioral experiments at P67. The animals were randomized and blinded to the investigators.

#### Open field test

The mice were habituated to the experimental room for 10 min before the experiment. Four dark Plexiglas boxes forming four square arenas (40 × 40 cm) were used.^81^ EthoVision XT 10 software (Noldus Information Technology, US) was used to define each arena. The mice were gently placed in the corner of each arena and video recorded for 10 min. All videos were recorded with a camera connected to the EthoVision system. The total distance covered and the average velocity within 10 min were calculated.

#### T-maze spontaneous alternation and novel object recognition tests

The T-maze was used to measure spatial working memory and recognition memory for 2 days. On day 1, the mice were placed into a T-maze consisting of 3 equally spaced arms (30 × 7 × 5 cm for each arm) made of black plastic (the arms were designated A, B, and C). The mice were allowed to explore the maze for 10 min in total, but only the first 3 min or 5 min (minimum of 12 arm entries) were used for analysis. Arm entries were classified into consecutive sequences of 3 arms (e.g., ACBCAC = ACB, CBC, BCA, CAC). An entry sequence was an alternation when it included A, B, and C in any order (e.g., BCA) but not when the same arm was entered more than once (e.g., CBC). The percentage of spontaneous alternations was calculated as follows:

Alternations (%) = (total number of arm entries - 2) ∗ 100

We re-analyzed the T-maze videos from day 1 for the locomotor activity. EthoVision XT 10 software (Noldus Information Technology, US) was used to track the moving distance for 10 min in total. Once mice were placed into the arm, EthoVision XT 10 started to track the mice and record the distance simultaneously. The mice stayed in the same arm for 10 min were excluded.

On day 2, two sessions of the novel object recognition test were performed. In the first session, a mouse was placed in a T-maze for 10 min and allowed to explore two identical Legos in the left and right arms. After 10 min, the mouse was removed from the maze and placed in its home cage for 3 min. One of the original Legos was replaced with a new one (with a different color and shape). The mouse was returned to the maze for another 10 min and allowed to explore both objects freely. The discrimination index (DI) was calculated as follows:

DI (%) =T_new_/(T_new_ + T_old_)∗100%

where T_new_ is the time spent exploring the new Lego, and T_old_ is the time spent exploring the old Lego. The videos were recorded with EthoVision XT 10 software (Noldus Information Technology, US).

#### Social preference

We used a three-chambered social box apparatus (40 × 60 cm), which was divided into 3 equal chambers (20 × 40 cm each) with square doors (4 × 4 cm). Two small acrylic cages were placed in the corners of the right and left chambers for the introduction of unfamiliar (intruder) mice. The sniffing zone was defined by the EthoVision XT 10 system (Noldus Information Technology, US). Each mouse was exposed to two consecutive sessions in the chambers: in the first session (adaptation), two empty acrylic cages were placed in the chambers, and a mouse was allowed to freely explore the three chambers for 10 min. After 10 min of habituation, the mouse was placed in the center zone with two closed doors.^76^ One intruder mouse of the same age and sex was placed in one of the acrylic cages, and the other acrylic cage was kept empty. Then, the doors were opened, allowing the mice to explore the three chambers for another 10 min. EthoVision XT 10 software (Noldus Information Technology, US) connected to an overhead camera was used to track the mice and record the amount of time spent in each of the sniffing zones. The location of the intruder mouse was changed across trials. Social preference (SP) was calculated as follows:

SP (%) = (T_intruder_)/(T_intruder_ + T_empty_)∗100%

where T_intruder_ is the time spent sniffing the zone containing the acrylic cage with the intruder and T_empty_ is the time spent sniffing the zone containing the empty acrylic cage.

#### Acoustic startle reflex and PPI

Startle reactivity was assessed in startle chambers (San Diego Instruments, San Diego, CA) controlled by SR-LAB software. The mice were placed on the platform for 5 min to allow habituation to 65 dB background noise. The startle session was divided into five blocks. In blocks 1 and 5, five 120 dB tones were delivered alone. In block 2, the reactivity of the mice reactivity to 80, 90, 100, 110, and 120 dB tones, which were delivered in random order. In block 3, PPI in response to 30 ms tones with an intensity of 3, 6, and 12 dB over background noise followed by a 50 ms 120 dB tone 100 ms later was evaluated. In block 4, the effect of the ISI on PPI was assessed. Tones with an intensity of 120 dB were presented alone or preceded by a 73 dB tone at a 25, 50, 100, 200, or 500-ms interval.^32^ The average startle magnitude (mV) through all the blocks was employed for data analysis. The PPI was calculated as a percentage according to the data obtained in block 3 by using the following formula:

PPI (%)=(100-(average startle magnitude in prepulse trial/average startle magnitude to pulse alone trial)∗100)

### Quantification and statistical analysis

The electrophysiological data were analyzed by using pClamp 10.2 software (Clampfit; Molecular Devices, US). All statistical analyses were performed in GraphPad Prism 8.0.2 (GraphPad Software Inc., San Diego, CA). The Kolmogorov-Smirnov test was used to analyze the cumulative probability. For two groups, the paired t test and unpaired t test were used. To compare multiple groups, one-way ANOVA (with or without repeated measures) and two-way ANOVA followed by the Dunnett test were used. The data are presented as the means ± SEMs. Sample sizes were chosen based on the value sufficient to observe statistical significance in prior studies. Statistical significance was determined when p∗<0.05; p∗∗<0.03; p∗∗∗<0.001, ns: not significant. All of the statistical details of the experiments can be found the figure legends.

## Data Availability

• This paper did not report new RNA sequence. Original western blot images have been deposited at Mendeley and are publicly available as of the date of publication. The DOI is listed in the key resources table. • This paper did not generate new codes. • Any additional information required to reanalyze the data reported in this paper is available from the lead contact upon request.
