# Supplementary material for: P2X7 purinergic receptor modulates dentate gyrus excitatory neurotransmission and alleviates schizophrenia-like symptoms in mouse
Source: iScience. 2023 Aug 7;26(9):107560. doi: 10.1016/j.isci.2023.107560 (PMC10462828; doi:10.1016/j.isci.2023.107560)
Supplement: Document S1. Figures S1–S4 and Tables S1–S4 [file mmc1.pdf]

iScience, Volume *26*

Supplemental information

P2X7 purinergic receptor modulates dentate gyrus excitatory neurotransmission and alleviates schizophrenia-like symptoms in mouse

Lumei Huang, Paula Mut-Arbona, Bernadett Varga, Bibiana Török, János Brunner, Antonia Arszovszki, András Iring, Máté Kisfali, E. Sylvester Vizi, and Beáta Sperlágh

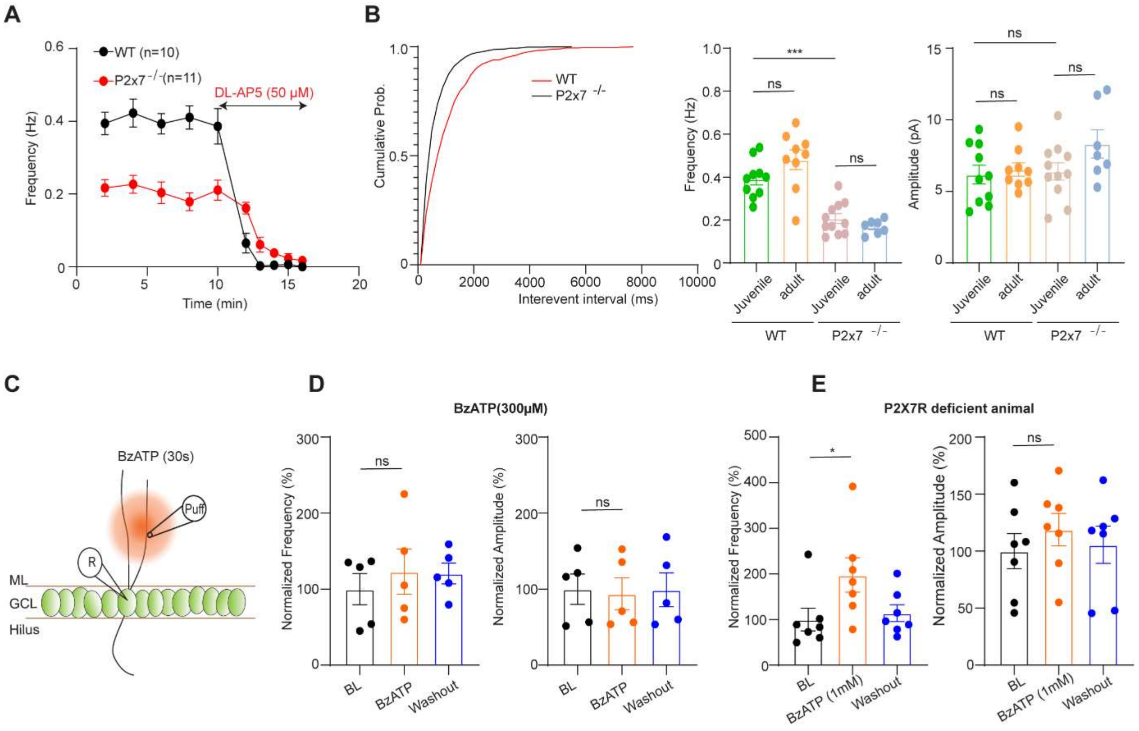

Figure S1 NMDA receptor-mediated mEPSC, related to Figure 1 and Figure 2. (A) The time course of NMDA receptor-mediated mEPSCs in DG GCs. DL-AP5 application abolished NMDA receptor-mediated mEPSCs in both WT and P2X7R deficient mice. (B) Cumulative probability of NMDA receptor-mEPSC interevent intervals in WT and P2X7R deficient at adult. Compared to WT, P2X7R deficient mice showed relatively lower number of events (Frequency WT: 0.48 ± 0.005 Hz, n=9 vs P2x7-/-: 0.16 ± 0.021 Hz, n=7; $p<0.0001$, unpaired t test) (Amplitude WT: 6.53 ± 0.47 pA, n=9 vs P2x7-/-: 8.31 ± 0.99 pA, n=7; p=0.98, unpaired t test). There was no difference between juvenile and adult (Frequency Juvenile: 0.39 ± 0.0027 Hz, n=10 vs adult: 0.48 ± 0.05 Hz, n=9; $p<0.0001$, one-way ANOVA by Dunnett's test). (C) The scheme for BzATP puff. (D) Application of 300 μM BzATP puff did not change the frequency (Normalized frequency (%): baseline: 100 ± 20.68; BzATP: 123.12 ± 29.80; Washout: 120.72 ± 13.70; n=5; baseline vs BzATP, p=0.69, one-way ANOVA repeated measures by Dunnett's test). (E) 1 mM BzATP puff increased the number of the NMDA receptor-mediated mEPSCs in P2X7R deficient mice (Normalized frequency (%): baseline: 100 ± 24.72; BzATP: 197.76 ± 37.74; Washout: 113.81 ± 18.28; n=7; baseline vs BzATP, p=0.04, one-way ANOVA repeated measures by Dunnett's test). The frequency and amplitude summary data are shown as the Mean ± SEM. * marks significant difference.

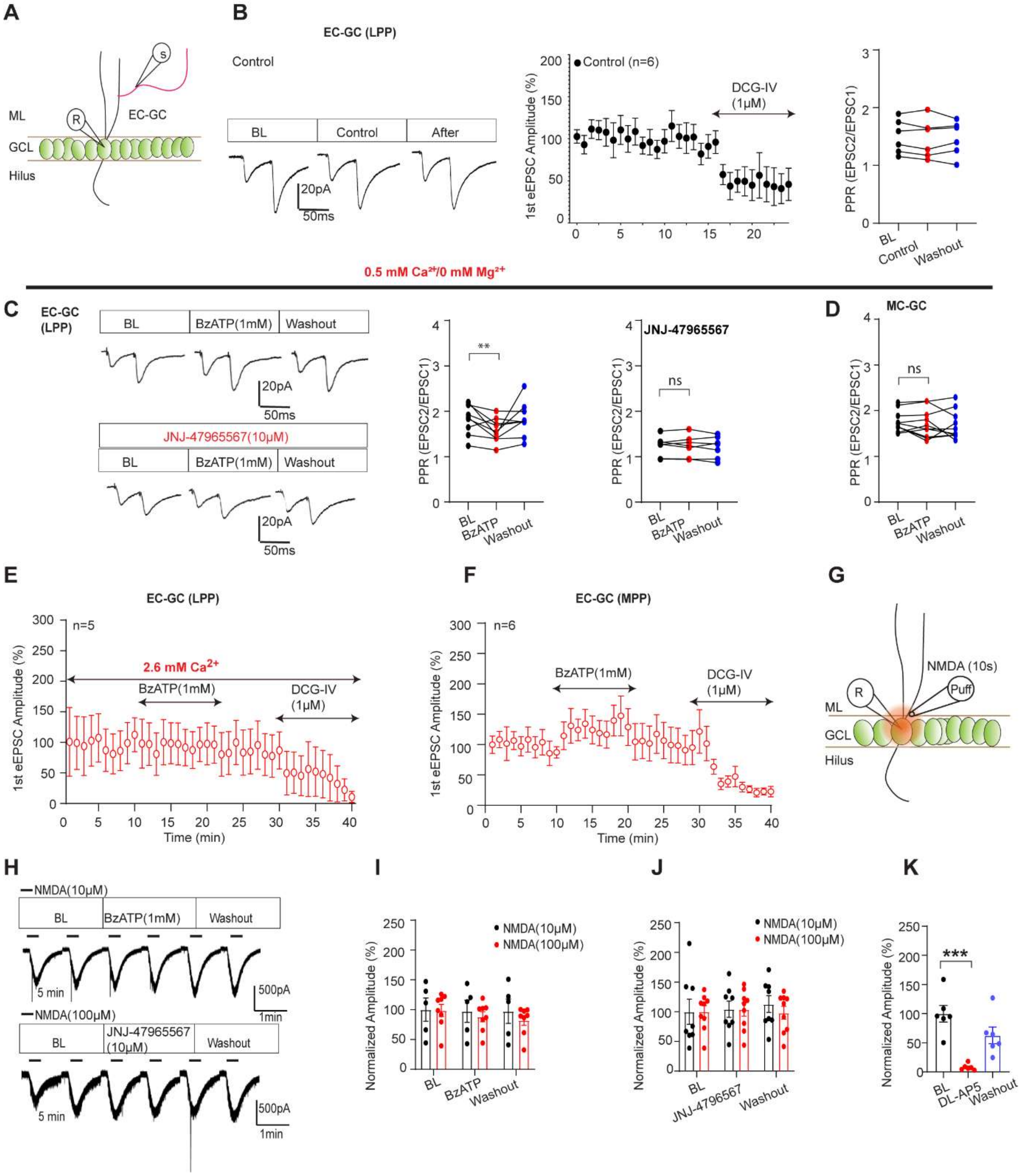

Figure S2 EC-GC and MC-GC stimulation, related to Figure 3. (A)Schematic of the stimulating and recording electrode. (B) The control recording in the presence of ARL47156 showing that ARL47156 alone did not alter PPR. (C, left) The original traces from EC-GC pathway recording in the presence of 0.5 mM Ca $^{2+}$/0 mM Mg $^{2+}$. (C, right) BzATP application decreased the PPR (PPR: baseline: 1.82 ± 0.12; BzATP: 1.57 ± 0.1; Washout: 1.83 ± 0.14; n=9; baseline vs BzATP, p=0.02, one-way ANOVA repeated measures by Dunnett's test), and JNJ-47965567 perfusion no longer changed the PPR (PPR: baseline: 1.23 ± 0.08; BzATP: 1.23 ± 0.81; Washout: 1.21 ± 0.9; n=7; baseline vs BzATP p=0.99, one-way ANOVA repeated measures by Dunnett's test). (D) MC-GC pathway recording in the presence of 0.5 mM Ca 2+/0 mM Mg 2+ and BzATP did not change the PPR in this pathway (PPR: baseline: 1.76 ±

0.07; BzATP: 1.71 ± 0.09; Washout: 1.68 ± 0.09; n=10; baseline vs BzATP, p=0.91, one-way ANOVA repeated measures by Dunnett's test). (E) BzATP-induced effect was blocked by using of 2.6 mM calcium in ASCF. (F) The 1st EPSC amplitude changes cross time in MPP. Compared to baseline, BzATP significantly potentiated the amplitude. (G) Schematic of the puff pipette and recording electrode. (H) Original traces induced by application of 10 NMDA via a pressure ejection system in the presence of BzATP and JNJ-47965567, respectively. (I) At 1 mM, BzATP did not change the 10 μM and 100 μM NMDARs-induced current amplitude relative to the baseline amplitude (Normalized current (%) for 10 μM: baseline: 100 ± 19.46; BzATP: 97.32 ± 18.57; Washout: 97.40 ± 20.43, n=5; Normalized current (%) for 100 μM: baseline: 98.73 ± 10.26; BzATP: 88.10 ± 8.51; Washout: 82.14 ± 8.37, n=8). (J) Inhibition of P2X7Rs by 10 μM JNJ-47965567 did not affect the amplitude of 10 μM and 100 μM NMDARs-induced currents (Normalized current (%) for 10 μM: baseline: 99 ± 21.12; BzATP: 104 ± 13.71; Washout: 112.9 ± 14.16, n=8; Normalized current (%) for 100 μM: baseline: 100 ± 10.09; BzATP: 103.76 ± 11.23; Washout: 98.28 ± 11.9, n=9). (K) Perfusion of the NMDA antagonist DL-AP5 dramatically inhibited NMDARs-induced currents (Normalized current (%): baseline: 100 ± 14.25; DL-AP5: 8.05 ± 2.22; Washout: 62.72 ± 14.30, n=6, BL vs DL-AP5, p=0.002, one-way ANOVA repeated measures by Dunnett's test). The data are presented as the Mean ± SEM. * marks significant difference.

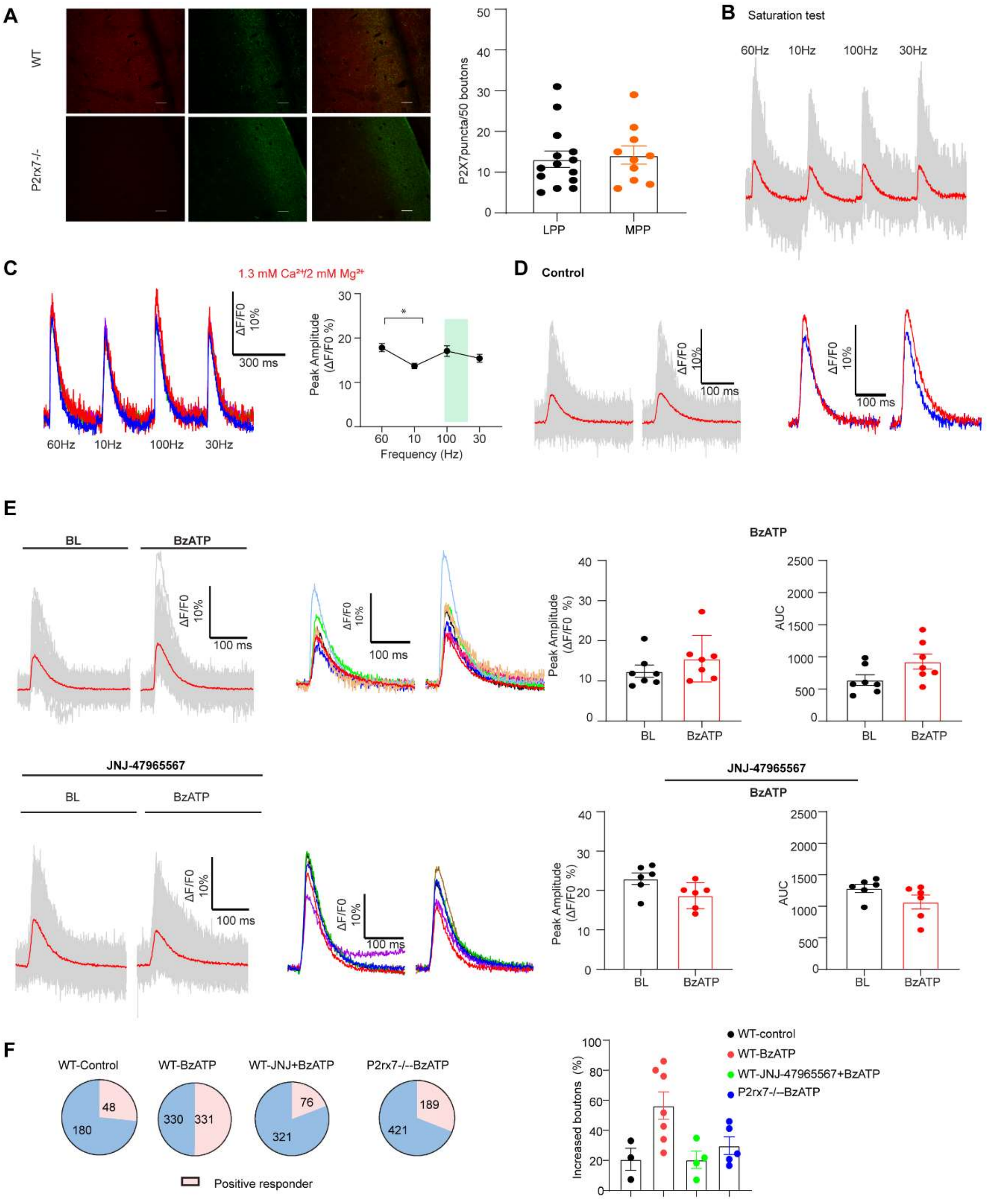

Figure S3 EC-GC axonal boutons calcium imaging, related to Figure 5. (A) The representative P2X7Rs immunostaining pictures in DG molecular layers in both WT and $P2x7^{-/-}$. The P2X7Rs positive puncta quantification results in LPP and MPP. The number of P2X7Rs positive puncta per 50 boutons in LPP did not differ from MPP (LPP (number): 13.21 ± 2.03 vs MPP (number):14.20 ± 2.23, unpaired t test, p=0.75). (B-C) pAAV1-hSynapsin1-axon-GCaMP6s saturation test. The calcium fluorescence intensity triggered by 5 electrical pulses at 10, 30, 60, and 100 Hz presented in random order. (B) The individual traces from one slice. (C, left) the average of 4 slices. (C, right) the summary data showing that 5 electrical pulses at 60 Hz led to saturation of the fluorescence intensity of this calcium indicator (60 Hz(%DF/F0): 17.8084 ± 0.92), 10 Hz: 13.65 ± 0.62；100 Hz: 17.05 ± 1.21; 30 Hz: 15.41 ± 0.89; n=4).

(D) The control recording showing the stability of the recording. (E) The original individual (left) and average traces(right) from BzATP application(upper) and JNJ-47965567(lower) in the presence of 0.5 mM Ca^{2+} /0 mM Mg^{2+}. BzATP significantly elevated both peak amplitude (BL(%DF/F0): 12.43 ± 1.31 VS BzATP (%DF/F0):15.54 ± 1.89, n=7, paired t test, p=0.01) and area under curve (BL (A.U): 639.8 ± 71.44 VS BzATP (A.U):926.71 ± 100.95, n=7; paired t test, p=0.56) (E upper). The increase was blocked by JNJ-47965567 (BL(%DF/F0): 22.76 ± 1.23 VS BzATP (%DF/F0):17.86 ± 1.14, n=6, paired t test, p=0.01; BL (A.U): 1286.03 ± 55.13 VS BzATP (A.U):1066.92 ± 93.66, n=6; paired t test, p=0.05). (F) The number and percentage of positively responding boutons showing an increase in the fluorescence density under different conditions. The fluorescence intensity was increased in 50% of boutons in response to BzATP application compared to 26% of boutons in response to control treatment, and this percentage of boutons that showed an increase in fluorescence intensity in response to BzATP was significantly higher than the percentages of boutons that showed an increase in fluorescence intensity in the presence of P2X7Rs antagonist JNJ47965567 (19%) and in P2X7R-deficient mice (30%)(WT-control: 20.73 ± 7.45%, n=3; WT-BzATP: 56.49 ± 9.15%, n=7; WT-JNJ-47965567+BzATP: 20.45 ± 5.74%, n=4; $P2x7^{-/-}$-BzATP: 29.78 ± 5.80%, n=5; WT-control vs WT-BzATP, p=0.028; WT-control vs WT-JNJ-47965567+BzATP, p>0.999; WT-control vs $P2x7^{-/-}$-BzATP, p=0.806, one-way ANOVA repeated measures followed by Dunnett's test). The data are shown as the mean ± SEM. * marks significant difference.

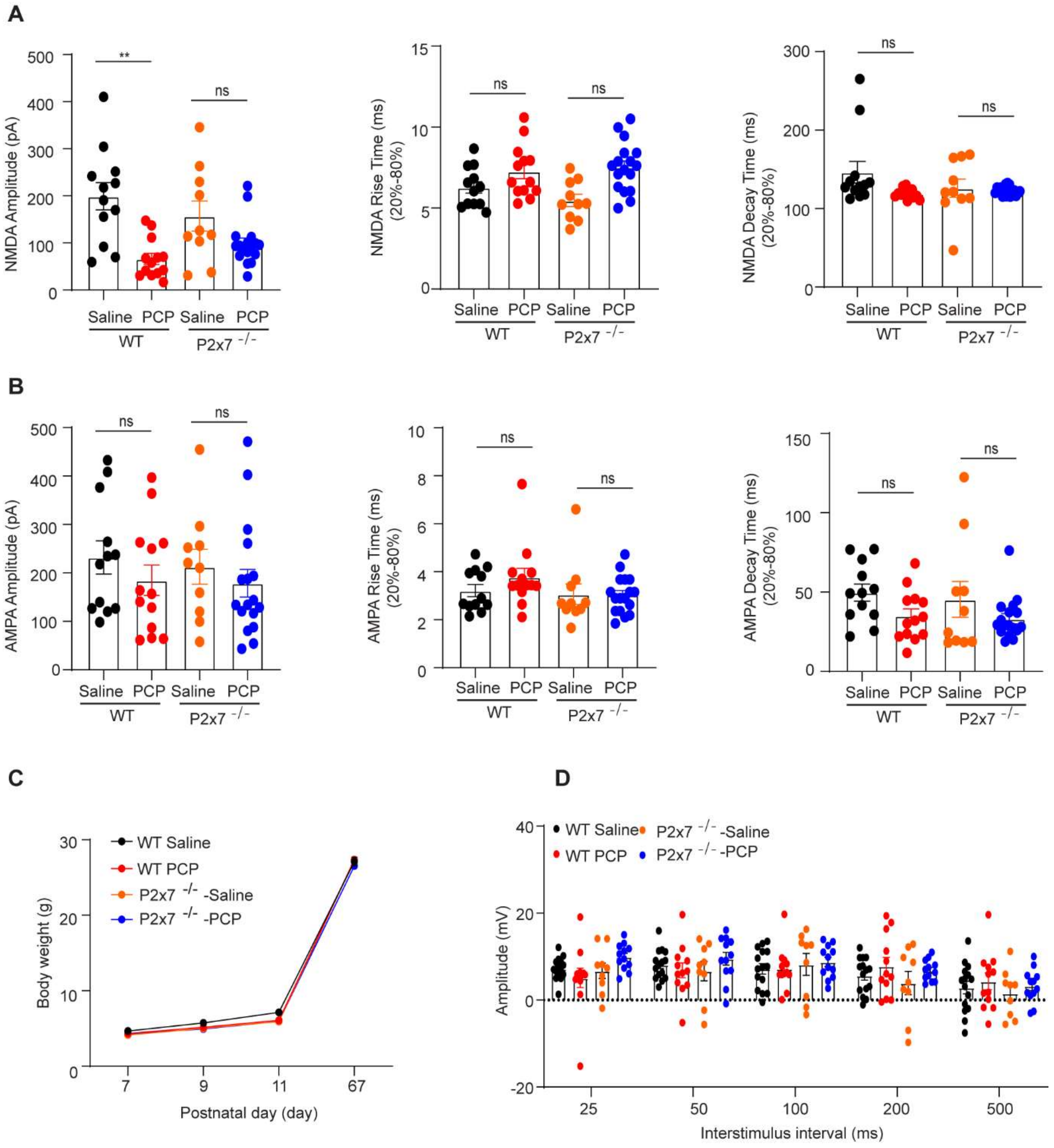

Figure S4 AMPA/NMDA ratio in different groups, related to Figure 6. (A and B) The AMPA/NMDA parameters under different conditions. (A) The amplitude of NMDARs currents decreased after PCP treatment in WT but not in P2X7R deficient groups (NMDA amplitude: WT-saline: 199.14 ± 29.11 pA, n=12 vs WT-PCP: 66.06 ± 11.56 pA, n=13, p=0.0002; P2x7$^{-/-}$-saline: 157.33 ± 31.88 pA, n=10 vs P2x7$^{-/-}$-PCP: 97.79 ± 11.44 pA, n=17, p=0.2072, one-way ANOVA by Dunnett's test). The rise time and decay time of NMDARs currents did not change in WT and P2X7R deficient mice treated with either saline or PCP. (B) No difference in the amplitude, rise time and decay time of AMPA currents in WT and P2X7R deficient mice treated with saline or PCP. (C) Body weight at different time points in different groups. No significant difference has been observed cross the groups at different time point. (D) The interstimulus interval in acoustic startle reflex did not change in different groups. Data shown as Mean ± SEM.

Figure	Frequency (Hz)			Amplitude (pA)		
	BL	BzATP	Washout	BL	BzATP	Washout
2C	0.43 ± 0.06	1.54 ± 0.20	0.39 ± 0.07	12.35± 1.75	13.7 ± 1.61	1.55 ± 1.48
	ANOVA, P< 0.001, n=6			ANOVA, P=0.97, n=6		
2D	0.55 ± 0.03	0.61 ± 0.06	0.53 ± 0.07	15.9 ± 3.03	16.64± 2.78	15.90± 2.53
	ANOVA, P= 0.73, n=9			ANOVA, p= 0.98, n=9		
2E	0.26 ± 0.03	0.94 ± 0.15	0.28 ± 0.3	9.34 ± 1.35	8.31 ± 0.59	8.77 ± 0.95
	ANOVA, P= 0.01, n=6			ANOVA, p= 0.97, n=6		
2F	0.27 ± 0.03	0.26 ± 0.03	0.22 ± 0.21	8.46 ± 0.69	8.90 ± 0.94	8.74 ± 0.83
	ANOVA, P= 0.73, n=8			ANOVA, p= 0.99, n=8		

Table S1 P2X7R activation induced NMDA receptor-mediated s/mEPSC, related to Figure 2. Data shown as Mean ± SEM. One-way ANOVA by Dunnett’s test.

	WT		Significance (ANOVA)	P2x7-/-		Significance (ANOVA)
	P20 (23)	P60 (20)		P20 (15)	P60 (14)	
RMP (mV)	-74.98 ± 1.26	-74.43 ± 1.11	P= 0.74	-74.26 ± 1.29	-73.71 ± 1.75	P= 0.79
IR (MΩ)	249.76 ±11.66	226.16± 14.81	P= 0.21	262.45 ±10.36	239.35 ± 8.67	P= 0.10
Maximal Freq (Hz)	31.69 ±1.03	31.55 ± 0.72	P= 0.91	30.56 ± 1.07	30.16 ± 0.86	P= 0.77
Threshold (mV)	-43.54 ± 1.71	-40.67 ± 1.29	P= 0.19	-42.58 ± 1.70	-40.70 ± 1.09	P= 0.37
Amplitude (mV)	78.00 ± 2.70	87.27 ± 2.62	p= 0.01	83.01 ± 2.40	87.00 ± 2.36	P= 0.24
Rise time (ms)	0.88 ± 0.020	0.85 ± 0.028	P= 0.87	0.88 ± 0.02	0.84 ± 0.03	P= 0.27
Half-width time (ms)	1.48 ± 0.03	1.48 ± 0.04	P= 0.62	1.49 ± 0.03	1.45 ± 0.05	P= 0.90
AHP (mV)	6.24 ± 0.36	9.78 ± 0.61	P < 0.0001	6.48 ± 0.29	8.94 ± 0.85	P= 0.01

Table S2 Membrane properties and intrinsic excitability of DG GCs in different groups, related to Figure 3. In WT groups, p60 mice only showed higher action potential amplitude and AHP amplitude compared to p20 mice. In P2x7$^{-/-}$ groups, no difference has been measured across all of the parameters, including RMP, IR, Maximal frequency. Threshold, amplitude, rise time, half-width time and AHP. Data shown as Mean ± SEM, statistical details (two-way ANOVA multiple comparison by Dunnett's test), cell number and p value have been showed in the table.

	WT		P2x7-/-	
	Saline (n=12)	PCP(n=13)	Saline (n=10)	PCP(n=17)
25 (ms)	35.74 ± 3.54	25.69 ± 12.26	35.41 ± 9.86	48.74 ± 4.64
	ANOVA, p>0.99		ANOVA, P=0.97	
50 (ms)	44.34 ± 4.67	35.52 ± 9.27	32.13 ± 12.58	45.47 ± 7.72
	ANOVA, p>0.99		ANOVA, P=0.97	
100 (ms)	34.15 ± 6.07	36.53 ± 7.63	38.32 ± 13.95	42.78 ± 5.77
	ANOVA, p>0.99		ANOVA, p>0.99	
200 (ms)	36.77 ± 5.43	40.37 ± 10.99	20.59 ± 15.14	33.06 ± 3.58
	ANOVA, p=0.99		ANOVA, p=0.96	
500 (ms)	10.77 ± 6.61	23.36 ± 10.37	5.10 ± 9.96	15.38 ± 6.03
	ANOVA, p>0.99		ANOVA, p>0.99	

Table S3 Startle reflex interstimulus interval in different groups, related to Figure 7. In both WT groups and P2x7-/-, PCP treated groups did not show difference in amplitude-induced by 25, 50, 100, 200 and 500 ms stimulus interval when compared to saline group. Data shown as Mean ± SEM, statistical details (two-way ANOVA multiple comparison by Dunnett’s test), animal number and p value have been showed in the table.

	WT		P2x7-/-	
	Saline (n=12)	PCP (n=13)	Saline (n=10)	PCP (n=17)
P7	4.66 ± 0.16	4.33 ± 0.14	4.14 ± 0.11	4.28 ± 0.07
	ANOVA, p=0.92		ANOVA, P=0.91	
P9	5.73 ± 0.21	6.08 ± 0.19	5.92 ± 0.16	5.59 ± 0.01
	ANOVA, p=0.74		ANOVA, P>0.99	
P11	7.13 ± 0.36	6.08 ± 0.19	5.92 ± 0.16	5.59 ± 0.01
	ANOVA, p>0.99		ANOVA, p>0.99	
P67	27.12 ± 0.57	27.35 ± 0.26	27.32 ± 0.41	26.54 ± 0.23
	ANOVA, p=0.99		ANOVA, p=0.93	

Table S4 Body weight at different time points in different groups, related to Figure 7 and Figure S2. Compared to saline injection, PCP treated groups in both WT and P2x7-/- mice did not show any difference at different time points. Data shown as Mean ± SEM, statistical details (two-way ANOVA multiple comparison by Dunnett's test), animal number and p value have been showed in the table.
